# Parameter identifiability analysis and visualization in large-scale kinetic models of biosystems

**DOI:** 10.1186/s12918-017-0428-y

**Published:** 2017-05-05

**Authors:** Attila Gábor, Alejandro F. Villaverde, Julio R. Banga

**Affiliations:** 1 BioProcess Engineering Group, IIM-CSIC, Eduardo Cabello 6, Vigo, 36208 Spain; 2 JRC-COMBINE, RWTH Aachen University, Photonics Cluster, Level 4, Campus-Boulevard 79, Aachen, 52074 Germany

**Keywords:** Parameter estimation, Dynamic models, Identifiability, Global optimization, Regularization, Overfitting

## Abstract

**Background:**

Kinetic models of biochemical systems usually consist of ordinary differential equations that have many unknown parameters. Some of these parameters are often practically unidentifiable, that is, their values cannot be uniquely determined from the available data. Possible causes are lack of influence on the measured outputs, interdependence among parameters, and poor data quality. Uncorrelated parameters can be seen as the key tuning knobs of a predictive model. Therefore, before attempting to perform parameter estimation (model calibration) it is important to characterize the subset(s) of identifiable parameters and their interplay. Once this is achieved, it is still necessary to perform parameter estimation, which poses additional challenges.

**Methods:**

We present a methodology that (i) detects high-order relationships among parameters, and (ii) visualizes the results to facilitate further analysis. We use a collinearity index to quantify the correlation between parameters in a group in a computationally efficient way. Then we apply integer optimization to find the largest groups of uncorrelated parameters. We also use the collinearity index to identify small groups of highly correlated parameters. The results files can be visualized using Cytoscape, showing the identifiable and non-identifiable groups of parameters together with the model structure in the same graph.

**Results:**

Our contributions alleviate the difficulties that appear at different stages of the identifiability analysis and parameter estimation process. We show how to combine global optimization and regularization techniques for calibrating medium and large scale biological models with moderate computation times. Then we evaluate the practical identifiability of the estimated parameters using the proposed methodology. The identifiability analysis techniques are implemented as a MATLAB toolbox called VisId, which is freely available as open source from GitHub (https://github.com/gabora/visid).

**Conclusions:**

Our approach is geared towards scalability. It enables the practical identifiability analysis of dynamic models of large size, and accelerates their calibration. The visualization tool allows modellers to detect parts that are problematic and need refinement or reformulation, and provides experimentalists with information that can be helpful in the design of new experiments.

**Electronic supplementary material:**

The online version of this article (doi:10.1186/s12918-017-0428-y) contains supplementary material, which is available to authorized users.

## Background

The development of mechanistic (kinetic) models in order to quantitatively describe the dynamics of biological phenomena is one of the core research themes in systems biology. During the last decade, fostered by the greater availability of the necessary experimental data, the development of large (up to genome-scale) kinetic models has become one of the main objectives in the field, as well as in related areas such as synthetic biology, metabolic engineering, or industrial biotechnology [1–10]. More recently, the first steps towards comprehensive whole-cell models have been taken [11], which has great potential for applications e.g. in personalized medicine [12]. However, the development of these large-scale integrated dynamic models poses severe challenges [13, 14]. Those associated with model building are common to the more general problem of reverse engineering of biological systems [15]. In this context, parameter estimation (i.e. model calibration) is arguably one of the most studied [16–19], yet more challenging step in model building.

Parameter estimation in nonlinear dynamic models can be an extremely hard problem mostly due to the following issues [15]: lack of identifiability, ill-conditioning, multimodality and over-fitting. The latter three can be handled via global optimization and regularization methods, as reviewed and illustrated recently [20]. The present paper begins by continuing the line of work in [20], addressing these three issues. To this end we introduce a combination of a global optimization metaheuristic, eSS [21], and an efficient local search method, the adaptive algorithm NL2SOL [22]. By using this optimization technique jointly with regularization it is possible to *reduce the calibration times* of large dynamic models and simultaneously avoid over-fitting. We show this for models from the recently presented BioPreDyn benchmark collection [23]. Then we focus on the remaining issue, that is, *identifiability analysis* of large dynamic models. Our aim is to develop a methodology which (i) is able to characterize high-order relationships among parameters, and (ii) scales up well with model size. Thus, our objective goes beyond finding the subset of identifiable parameters: we also aim to systematically characterize the space of non-identifiable parameters, and to facilitate the advanced analysis of the results with scalable visualization tools.

Identifiability analysis aims at establishing whether it is possible to determine the values of the unknown model parameters [24]. It is common to distinguish between structural and practical identifiability. *Structural* or a priori identifiability analysis decides whether the model parameters are uniquely determinable based on the model formulation, which includes the dynamic equations, observation functions and stimuli [25]. A parameter *θ* of the model is structurally identifiable if *y*(*θ*)=*y*(*θ* ^′^)⇔*θ*=*θ* ^′^, where *y* denotes the model predictions, which are observable in the experiments. A parameter *θ* is structurally *locally* identifiable if for almost any value *θ* ^∗^ there is a neighbourhood *V*(*θ* ^∗^) in which the above relationship holds. It is *globally* identifiable if the relationship holds in all the range of values of the parameter. If there is some region with non-zero measure where the relationship does not hold, *θ* is structurally unidentifiable. Structural identifiability analyses usually involve a high computational burden, which makes them difficult to apply to large models [26–28]. Furthermore, structural identifiability is only a necessary but not sufficient condition for identifiability. Very often a structurally identifiable parameter is practically unidentifiable, that is, its value cannot be determined with precision due to limitations in the available data. This can be quantified using *practical* or *a posteriori* identifiability analysis, which provides confidence intervals of the parameter values. The two main sources of practical non-identifiability are (1) lack of influence of a parameter on the observables, and (2) interdependence among the parameters. Obviously, if a parameter does not influence the observables (case 1) it is not possible to determine its value. The second situation, in which the effect on the observables of a change in one parameter can be compensated by changes in other parameters, can also prevent parameter identification. Both problems are related to the sensitivities of the observables to changes in model parameters. While (1) is related to the average sensitivity of the model outputs to a specific parameter, (2) can be investigated based on the collinearity of the parametric sensitivities [29].

In this paper we combine global optimization and regularization techniques to calibrate medium and large scale biological models (in this context, we will use the term “medium-scale” for models with 10 to 50 parameters and “large-scale” for models with more than 50 parameters). Then we evaluate the practical identifiability of estimated model parameters using sensitivity analysis and collinearity measures. We determine the largest identifiable subsets of parameters, characterize the interplay among non-identifiable groups of parameters, and visualize the results using Cytoscape. The visualization tool shows the identifiable and non-identifiable groups of parameters together with the model structure in the same graph. In this way, modellers can detect parts that are problematic and need refinement or reformulation, and experimentalists obtain information that can be helpful in the design of new experiments. The methods for identifiability analysis and visualization presented here have been implemented as a MATLAB toolbox called VisId, which is available from GitHub (https://github.com/gabora/visid) and as Additional file 1.

## Methods

### Parameter estimation with regularization and global optimization

#### Mathematical model

We consider deterministic models of biological systems that can be described by nonlinear ordinary differential equations (ODEs) in the following form:

1 $$\begin{array}{*{20}l}  \frac{dx(t,\theta)}{dt} &= f(x(t,\theta),u(t),\theta), \end{array} $$

2 $$\begin{array}{*{20}l}  y(x,\theta) &= g(x(t,\theta),\theta), \end{array} $$

3 $$\begin{array}{*{20}l}  x(t_{0}) & = x_{0}(\theta), \quad t \in \left[t_{0},\,t_{f}\right]. \end{array} $$

Here $x \in \mathbb {R}^{N_{x}}$ denotes the state vector (often concentrations), *f* describes the interactions among the state variables (often constructed from the reaction rate functions), and *u*(*t*) denotes the input variables (stimuli). The parameter vector $\theta \in \mathbb {R}^{N_{\theta }}$ contains the (positive) parameters, e.g. reaction rate coefficients or Hill exponents. Their values are often unknown and must be estimated from data.

The model variables *x* are mapped to the measurable output variables $y \in \mathbb {R}^{N_{y}}$, also known as observables or model predictions, by the observation function *g*. These *y* signals are the quantities that can be experimentally measured. We will denote by *y*_*ijk*_ the model prediction for the *j*-th observed quantity in the *k*-th experiment at time *t*_*i*_∈[*t*_0_, *t*_*f*_]. The corresponding measured data is denoted by $\tilde {y}_{ijk}$.

#### Parameter estimation

The goal of parameter estimation is to determine the values of the unknown parameter vector *θ*. This is usually done by minimizing a distance between model prediction *y*_*ijk*_ and measured data $\tilde {y}_{ijk}$. One of the simplest, but yet general, choices of this distance is the weighted sum-of-squares

4 $$  Q_{\text{LS}}(\theta) = \sum\limits_{k=1}^{N_{\mathrm{e}}} \sum\limits_{j=1}^{N_{y,k}} \sum\limits_{i=1}^{N_{t,k,j}}w_{ijk}\left(y_{ijk}\left(x(t_{i},\theta),\theta\right)-\tilde{y}_{ijk}\right)^{2},  $$

where *N*_e_ is the number of experiments, *N*_*y*,*k*_ is the number of observed compounds in the *k*-th experiment, and *N*_*t*,*k*,*j*_ is the number of measurement time points of the *j*-th observed quantity in the *k*-th experiment, and the weights are denoted by *w*_*ijk*_. The total number of data in all experiments is denoted by $N_{D} = \sum _{k=1}^{N_{\mathrm {e}}} \sum _{j=1}^{N_{y,k}} \sum _{i=1}^{N_{t,k,j}} 1$. In order to simplify the index triplet, from now on we will use only one index, i.e. the weights and observables are denoted by *w*_*i*_ and *y*_*i*_ for *i*=1, 2…*N*_*D*_.

Then the parameter estimation problem is formulated as an optimization problem in the following form:

5 $$\begin{array}{*{20}l}  \underset{\theta}{\text{minimize}} &~ Q_{\text{LS}}(\theta)+\alpha \Gamma(\theta) \end{array} $$

6 $$\begin{array}{*{20}l}  \text{subject to } & \theta_{\text{min}} \leq \theta \leq \theta_{\text{max}}, \end{array} $$

7 $$\begin{array}{*{20}l}  & \frac{dx(t,\theta)}{dt} = f(u(t),x(t,\theta),\theta), \end{array} $$

8 $$\begin{array}{*{20}l}  & y(x,\theta) = g(x(t,\theta),\theta), \end{array} $$

9 $$\begin{array}{*{20}l}  & x(t_{0}) = x_{0}(\theta), \quad t \in \left[t_{0},\,t_{f}\right]. \end{array} $$

Here *Γ*(*θ*) is a a regularization term, which is described in the following subsection, and *θ*_min_ and *θ*_max_ are lower and upper bounds of the parameter values. The parameter vector $\hat \theta $ that solves this minimization problem is called the *optimal parameter vector* or the parameter estimates.

#### Regularization

Large scale dynamic models are often over-parametrized, turning the estimation of their parameters into an ill-posed problem [30]. This means that the minimum of the least-squares cost function (4) is non-unique, or that even a very small perturbation of the data results in very different estimated parameters. Furthermore, due to the large number of degrees of freedom, these models tend to capture the artificial dynamics of measurement noise. This is known as overfitting [31, 32] and it usually results in poor predictive capability of the calibrated model.

Regularization techniques incorporate a priori knowledge about the parameter values to make the problem well-posed. The regularization parameter *α* in (5) balances the strength of this knowledge; its value can be found by regularization tuning methods [33]. Here we followed the guidelines presented in [20] and chose a small regularization parameter (*α*=0.1), since we assume that we do not have good a priori estimates of the parameters.

Regarding the regularization function, *Γ*(*θ*), we chose the Tikhonov regularization framework to match the form of the penalty to the least squares formalism of the objective function. In this case the penalty is a quadratic penalty function,

10 $$  \Gamma(\theta) = \left(\theta-\theta^{\text{ref}}\right)^{T}W^{T}W\left(\theta-\theta^{\text{ref}}\right),  $$

where $W\in \mathbb {R}^{N_{\theta } \times N_{\theta }}$ is a diagonal scaling matrix and $\theta ^{\text {ref}}\in \mathbb {R}^{N_{\theta }}$ is a reference parameter vector, which is problem dependent and determined by the available information about the model parameters.

#### Global optimization

We solve the minimization problem defined by (5)–(10) using optimization. Since the cost function (5) is usually multi-modal (i.e. it usually has several local minima) [34–37], it is necessary to use an efficient global optimization method. Deterministic global optimization methods [38–42] can guarantee global optimality of the solution. However, their computational cost increases exponentially with the number of parameters, which makes them unsatisfactory for large scale models. Stochastic and metaheuristic methods [17, 18, 35, 36, 43, 44], on the other hand, do not provide such guarantees, but are often capable of finding adequate solutions in reasonable computation times.

For this reason we use a method called enhanced scatter search (eSS) [21], which is an advanced implementation of a population-based algorithm called scatter search. The scatter search metaheuristic works by evolving a number of solutions (population members), which constitute the reference set (RefSet). Members of this set are selected due to their quality and diversity. They are updated at every iteration by combining them with other RefSet members and, occasionally, by applying an improvement method. This improvement consists of a local search to speed-up the convergence to optimal solutions. In the present work we have chosen NL2SOL [22] as a local method. NL2SOL is a quasi-Newton algorithm with trust region strategy that exploits the structure of the nonlinear least squares problem. Note that the combination of a global method (scatter search) with a local one makes eSS a hybrid algorithm.

### Practical identifiability analysis

The shape of the cost function (5) in the surroundings of its optima determines the local identifiability of the parameters. We assess parametric identifiability in two consecutive steps:

1. First we calculate the sensitivity of the model outputs (observables) with respect to changes in the parameters. Those parameters which have no effect (or very little) on the observed signals are classified as non-identifiable. Note that this label is assigned on an individual basis, that is, taking only into account the effect of each parameter individually.
2. Even if a parameter influences the model output, it may still be unidentifiable if its effect can be compensated by changes in other(s) parameter(s). Hence in the second step we consider the interplay among parameters, aiming at finding groups of parameters which are non-identifiable due to their collinearity.

Note that, while it would be possible at least in principle to perform both steps simultaneously, in practice the curse of dimensionality hampers the application of such a global sensitivity approach to large models [45, 46].

#### Sensitivity analysis

The analysis of parametric sensitivity of kinetic models has a long tradition in model analysis [47, 48]. For the dynamical system (1)–(2), the parametric sensitivities of the observables can be accurately calculated by solving the forward sensitivity equations:

11 $${} \frac{dX_{i}(t)}{dt} = \frac{\partial f(x,u,\theta)}{\partial x}X_{i}(t) + \frac{\partial f(x,u,\theta)}{\partial \theta} \quad\text{for } i = 1,\dots, N_{\theta}  $$

12 $${} s_{i}(t) = \frac{\partial g(x,\theta)}{\partial x}X_{i}(t) + \frac{\partial g(x,\theta)}{\partial\theta} \quad\text{for } i = 1,\dots, N_{\theta}  $$

13 $${} {{s_{i}(t_{0}) =\left\{ \begin{array}{ll} 0 & \text{if } \theta_{i} \text{ is a model parameter}\\ 1 & \text{if } \theta_{i} \text{ is an initial condition} \end{array}\right. \quad\text{for } i = 1,\dots, N_{\theta}. }}  $$

Here $X_{i}=\frac {\partial x}{\partial \theta _{i}}$ denotes the *sensitivity of the state vector* with respect to the *i*-th parameter and the vector $s_{i} = \frac {\partial y}{\partial \theta _{i}}$ is the *sensitivity of the observables* with respect to this parameter. This calculation requires the solution of the *N*_*x*_×*N*_*θ*_ ordinary differential Eq. (11) with initial conditions (13) for each experiment. The numerical solution is determined for the time points for which there are experimental data available, and then the algebraic Eq. (12) are evaluated. If the partial derivatives of the dynamic equations are not available, an alternative is to calculate the sensitivities using finite differences or automatic differentiation.

The sensitivities of the observables are scaled using the same weights as in Eq. (4), resulting in scaled sensitivities for an output *j* and a parameter *i*:

14 $$  \left[\tilde{s}_{i}\right]_{j} =\sqrt{w_{j}}\frac{\partial y_{j}}{\partial \theta_{i}}.  $$

For each parameter we calculate an overall scoring called root mean squared sensitivity, $\tilde {s}_{i}^{\text {msqr}}$, to take into account changes in time or across experiments [29, 49]:

15 $$  \tilde{s}_{i}^{\text{msqr}} = \sqrt{\frac{1}{N_{D}}\sum\limits_{j=1}^{N_{D}}\tilde{s}_{ij}^{2}} ~~ \text{for} ~~ i = 1,\dots, N_{\theta}~~.  $$

Below a certain threshold the parameters are considered non-influential to the outputs. We set the threshold to four orders of magnitude smaller than the maximum root mean square value (15). Parameters whose sensitivity falls below this cut-off value are considered practically non-identifiable and they are kept out of further analysis. The procedure is summarized in Algorithm 1.



We remark that the outcome of the sensitivity calculations depends not only on the parameters, but also on the choice of initial conditions and external stimuli, which can have a strong influence in the practical identifiability of a model. If insufficiently excitatory stimuli or initial conditions result in poor practical identifiability, a solution – if it is possible to carry out additional measurements – is to design and perform a new experiment to generate maximally informative data [17].

#### Collinearity of parameters

Interplay among influential parameters can result in an unidentifiable model, because a variation in the cost function value due to a change in a parameter can be compensated by changes in other parameters. Pairwise interplay can be detected by plotting contours of the cost function versus pairs of parameters. Largely eccentric contours or “valleys” show that the cost function is almost unchanged in one direction, and the two parameters are highly correlated. This approach has two drawbacks: it involves a large computational effort and is limited to interplay between pairs of parameters. To compute higher dimensional interactions we use a different measure: the *collinearity* of parametric sensitivities.

To calculate collinearity we first normalize the scaled sensitivities (14) as follows:

16 $$  \bar{s}_{i} = \frac{\hat{s}_{i}}{\|\hat{s}_{i}\|} ~~ \text{for}~~i=1,\dots, N_{\theta}.  $$

This normalization avoids biases caused by differences in the absolute values of the individual sensitivity vectors.

Let us consider a set *K* of *k* parameters and their corresponding sensitivity vectors. The parameters are linearly dependent if there exist *k* constants *α*_*i*_≠0 such that

17 $$  \alpha_{1} \bar{s}_{K_{1}} + \alpha_{2} \bar{s}_{K_{2}} + \dots \alpha_{k} \bar{s}_{K_{k}} = 0  $$

If the above relation does not hold, the set is independent. When the equality (17) holds only approximately, the parameters are nearly dependent or nearly collinear. The degree of collinearity among a set of parameters can be measured by the collinearity index, CI_*K*_, which is defined as [29]:

18 $$ \text{CI}_{K} = \frac{1}{\min_{\|\alpha\| = 1}\|\bar{S}_{K}\alpha\|}=\frac{1}{\sqrt{\lambda_{K,\text{min}}}}.  $$

where $\bar {S}_{k}$ is the sensitivity matrix built from the *k* sensitivity vectors, $\bar {S}_{K}=[\bar {s}_{K_{1}},\bar {s}_{K_{2}}\dots \bar {s}_{K_{k}}]$, and *λ*_*K*,min_ is the smallest eigenvalue of $\bar {S}_{K}^{T}\bar {S}_{K}$. The larger the collinearity index is, the more dependent the corresponding parameters are. Brun and co-authors [29] proposed to classify a subset of parameters as identifiable if their collinearity index is smaller than a threshold which they chose as CI_*K*_<20. Roughly speaking, a value of 20 means that 95% of the variation in the model output caused by changing one of the parameters in the subset can be compensated by changing the other parameters in the set.

Other approaches for finding parameter correlations using sensitivity-based measures have been previously proposed in the literature. Li and Vu presented two methods [50, 51] that search for relationships among parameters in the context of a priori identifiability analysis (i.e. with noise-free, continuous data). The method in [50] provides a necessary but not sufficient condition for identifiability of nonlinear systems, which need to be fully observed (i.e. they must satisfy *y*=*x*). The method in [51] removes the requirement of measuring all the system states, replacing it with the restriction that the model must be linear. We remark that the method proposed in the present manuscript does not have these limitations: it can be applied to partially observed, nonlinear systems with noisy, discrete-time measurements.

#### Largest identifiable subset

As explained in the previous subsections, a subset of parameters is considered identifiable if its elements are influential and their sensitivity vectors are not collinear. We are interested in finding the largest set of parameters for which the collinearity of the corresponding sensitivity vectors is below the chosen threshold, CI_*K*_<20. Such a set of parameters represents all the degrees of freedom in the model. This means that perturbing a parameter *not* included in this set has an effect in the model predictions that can be compensated (at least by 95%) by changing other parameters in the set. However, a perturbation in a parameter belonging to the set cannot be compensated by changes in the remaining parameters.

Several methods have been developed for finding the group of identifiable parameters [30, 52, 53]. *Iterative* selection methods apply a step-wise procedure to select one parameter at a time, until no more parameters can be added to the identifiable set. In each step the parameter to be included is selected based on an optimality criteria. For example, the modified Gram-Schmidt orthogonalization method [54] projects all the remaining sensitivity vectors to the subspace spanned by the already selected sensitivity vectors, and includes the parameter corresponding to the one with the largest projection value. This step is repeated until the largest projection value falls below a threshold, which means that the next parameter would significantly interplay with the parameters already included. The computational cost of this method scales up well with the number of sensitivity vectors. However, the drawback of iterative procedures such as this one is that the solution might not be the global optimum, that is, it might fail to find the largest identifiable subset.

Alternatively, we propose to solve the problem of finding the largest identifiable subset of all the estimated parameters using *combinatorial optimization*. To this end we formulate it as a (nonlinear) integer optimization problem, where the goal is to maximize the number of sensitivity vectors included in the set, with the constraint that the corresponding collinearity index is below a threshold CI^∗^.

This algorithm can be stated as

19 $$\begin{array}{*{20}l} \underset{i\in \{0,1\}^{N_{\theta}}}{\text{maximize}} &~ \sum\limits_{k=1}^{N_{\theta}} i_{k} \end{array} $$

20 $$\begin{array}{*{20}l} \text{subject to}\! &~~S_{i} \!= \!\text{cat}\!\left(\{s_{k}~\!|\!~i_{k}\,=\,1,~\text{for} ~k=1,\dots, N_{\theta}\}\right) \end{array} $$

21 $$\begin{array}{*{20}l} &~~\text{CI}(S_{i})<\text{CI}^{*} \end{array} $$

22 $$\begin{array}{*{20}l} &~~i_{k} \text{ is a binary variable for }k=1,\dots, N_{\theta} \end{array} $$

where the binary variable *i*_*k*_ indicates if the *k*-th parameter is included (*i*_*k*_=1) or not included (*i*_*k*_=0) in the identifiable group of parameters. The sensitivity matrix corresponding to the selected parameters is *S*_*i*_, and ‘cat’ stands for the concatenation of the column vectors in the constraint (20). The collinearity index of this matrix is CI(*S*_*i*_) and it is determined by computing the minimum eigenvalue as in (17).

This combinatorial optimization problem has an exponentially scaling computational cost, and thus its solution requires an efficient algorithm. We chose the Variable Neighbourhood Search (VNS) technique [55], which is a heuristic global optimization method for integer optimization problems. We used the version of VNS included in the MEIGO Toolbox [56], which is implemented in MATLAB.

We modified this initial formulation of the problem described in Eqs. (19)–(21) after finding that its solution is often not unique: even after maximizing the number of parameters in the subset, there may be multiple subsets that yield a collinearity index below the threshold CI^∗^. Indeed, we found large variability in the solutions if no initial guess was specified. Therefore, we reformulated the optimization problem in two ways, as described in the following paragraphs.

As a first modification, we transformed the collinearity requirement (21) from a ‘hard’ to a ‘soft’ constraint (or penalty). The modified optimization problem reads as

23 $$\begin{array}{*{20}l} \underset{i\in \{0,1\}^{N_{\theta}}}{\text{maximize}} & \sum_{k=1}^{N_{\theta}} i_{k} - P_{1}(i)- P_{2}(i) \end{array} $$

24 $$\begin{array}{*{20}l} \text{subject to} & ~~S_{i} = \text{cat}\left(\left\{s_{k}~|~i_{k}=1,~\text{for}~ k=1,\dots, N_{\theta}\right\}\right) \end{array} $$

25 $$\begin{array}{*{20}l} & ~~P_{1}(i) = \frac{1}{2} \text{CI}(S_{i})/\text{CI}^{*}  \end{array} $$

26 $$\begin{array}{*{20}l} &~~ P_{2}(i) =\left\{\begin{array}{ll} \!\!0 & \!\text{if}~~\text{CI}(S_{i}) < \text{CI}^{*}\\ \!\!\alpha\left(\text{CI}(S_{i}) - \text{CI}^{*}\right)^{\beta} &\! \text{otherwise} \end{array}\right.  \end{array} $$

27 $$\begin{array}{*{20}l} &~~i_{k} \text{ is a binary variable for }k=1,\dots, N_{\theta} \end{array} $$

As above, the binary variable *i*_*k*_ indicates if the *k*-th parameter is included (1) or not included (0) in the selected group of parameters. The penalty *P*_1_ is a monotone increasing (linear) function of the collinearity index CI(*S*_*i*_), such that *P*_1_ is 0.5 when the collinearity equals to the threshold. Due to this small value, *P*_1_ does not influence the size of the largest subset below the threshold. In this way, when multiple sets of the same size co-exist, the set with smaller collinearity index is always favoured. This results in an unique solution of the optimization problem if there are no sets with identical collinearity index. The second penalty function *P*_2_ represents a soft constraint that is active when the collinearity exceeds the threshold. The steepness of this constraint is tuned by the values of *α* and *β*, which we set to *α*=1 and *β*=2.

Our second improvement of the formulation of the optimization problem consists in providing a good initial guess of the solution using QR decomposition. The rank revealing QR decomposition algorithm, or rrqr [57], rewrites a matrix *S* as

28 $$ \Pi S = QR,  $$

where *Q* is an orthogonal matrix, *R* is an upper triangular matrix, and *Π* is a permutation matrix. Due to the properties of this decomposition, the permutation matrix defines a reordering of the columns of *S*. In this re-ordered matrix *S*_ro_=*Π* *S*, the most orthogonal columns are located in the left. In other words, the first *n* columns of the reordered matrix define a linear subspace, and the (*n*+1)-th column has the largest projection value on this subspace among the remaining *N*_*θ*_−*n* columns located to the right of the *n*-th column. The outcome of the rrqr technique is similar to that of the aforementioned Gram-Schmidt orthogonalization method, but its implementation is more efficient.

We applied rank revealing QR decomposition to the sensitivity matrix, following the procedure described in Algorithm 2. Then, we used the resulted ordering of the sensitivity vectors to initialize the global optimizer. In this way we improved the performance of the global optimizer, which often found larger sets with collinearity index below the threshold value. The whole procedure for identifying the largest non-collinear subset of parameters is summarized in Algorithm 3.





The procedure presented in this subsection has similarities with the one proposed by Chu and Hahn [54]. One difference is that we maximize the subset size for a given collinearity threshold, whereas Chu and Hahn adopted the opposite approach, i.e., maximizing parametric identifiability for a pre-specified subset size. Additionally, both methods differ in the optimization technique: we use Variable Neighbourhood Search, which has better scalability than the genetic algorithm chosen in [54]. Recently, Nienałtowski et al. [58] have proposed a method for finding clusters of correlated parameters using so-called canonical correlation analysis (CCA). CCA is an extension of Pearson correlation for measuring multidimensional correlations between groups of parameters. Given two groups of parameters of sizes *m* and *n*, with *m*<*n*, calculation of the canonical correlations provides *m* measures, which are summarized in a single measure, called MI-CCA. This similarity measure represents the mutual information between the two groups, although it should be noted that average mutual information is equivalent to canonical correlation only if the random variables follow an elliptically symmetric probability model. Nienałtowski et al. use MI-CCA to cluster parameters until an identifiable subset is reached. This approach is sequential and yields a single parameter subset, which is possibly not maximal. In contrast, the methodology described here combines an initial sequential phase with a subsequent combinatorial optimization procedure. The second phase yields several identifiable parameter subsets and usually improves the initial solution.

#### Finding all largest subsets

As mentioned above, the largest non-collinear subset of parameters is not unique. To realize this, imagine that we have a non-collinear set of the parameters, and consider an additional pair of highly collinear parameters. Since we may add either of these two parameters to the set, but not both of them, we have two potential solutions. The optimization algorithm described above would choose the option with a lower collinearity index.

However, we may also be interested in enumerating *all* the possible sets, instead of only one. Finding all the largest subsets is a combinatorial problem too, which is computationally expensive. A naive approach for solving it could be to generate all possible sets of parameters and compute the corresponding collinearity index. However, note that if two parameters *θ*_1_ and *θ*_2_ are collinear, then any sets including the pair { *θ*_1_,*θ*_2_} are highly collinear. Using this fact, we developed an incremental procedure for the systematic determination of the sets. We start by considering all possible pairs of parameters and determining their collinearity. Then we extend only those pairs which have a small collinearity index, by considering all possible combinations of a third parameter. This procedure is repeated until either all the sets are highly collinear, or there is only one set containing all the parameters. In this way, summarized in Algorithm 4, we can find all the largest subsets of non-collinear parameters.



#### Partitioning the non-identifiable parameters

The two procedures presented above can be used for finding (i) the largest, least collinear subset of parameters, and (ii) all the largest subsets; in both cases, restricted to those subsets whose collinearity falls below a threshold. However, it is often important to understand why certain parameters are *not* identifiable. For example, a parameter may be unidentifiable because the model output has very low sensitivity to changes in its value. But it could also be because it is highly correlated with another parameter, even when both parameters have high sensitivities. Finding small groups of highly collinear parameters can be helpful in determining the exact source of unidentifiability.

The collinearity of a subset always increases when a new parameter is added to the set. For example, considering three parameters, the collinearity of the triplet is always higher than the collinearity of any pairs. Therefore, if a larger set of parameters contains a collinear pair, then the collinearity index of the large set is also large.

If we are interested in *finding the smallest groups of highly collinear parameters*, we can proceed as follows. First we generate all possible pairs of parameters, and compute the collinearity of the corresponding sensitivity vectors. Then we evaluate all possible triplets. The procedure can be extended for the analysis of larger sets. However, due to the combinatorial explosion of the computational cost, this method can be applied only to models of moderate size (with a maximum of roughly 20 parameters).

### Visualization of identifiable subsets

It can be useful to represent the identifiability results graphically, because such visualization can provide modellers with insight about how to reformulate their models and/or design new experiments in order to avoid non-identifiable parameters.

**Fig. 1:**
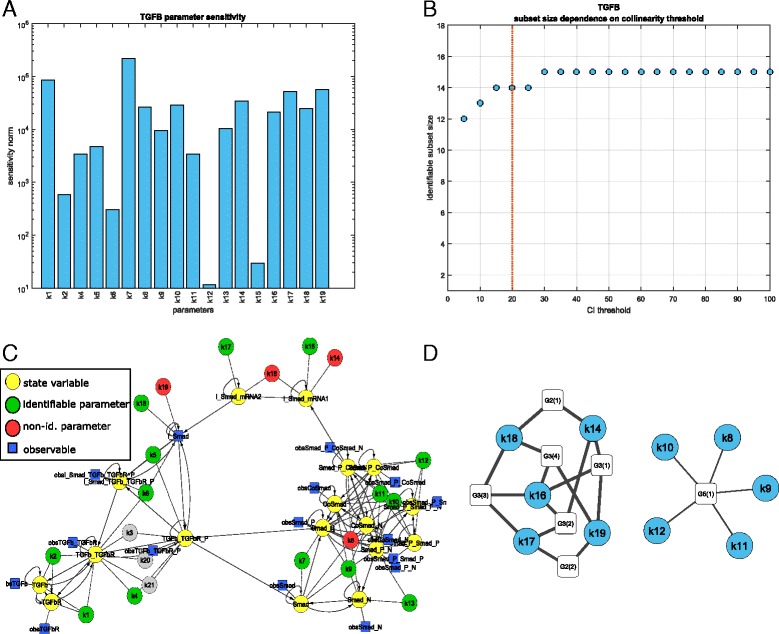
TGF- *β* model: panel **a** shows the model sensitivities with respect to the parameters in logarithmic scale. In panel **b** the maximum number of identifiable parameters is depicted depending on the collinearity threshold. The group-size does not change much by the threshold. The *red vertical line* indicates our choice (CI =20) for the further analysis. In panel **c** the mathematical model is depicted. Nodes indicate states (*yellow*), identifiable (*green*) and not identifiable (*red*) parameters, and observables (*blue*). In panel **d** the interplay among the collinear parameters are indicated. There are 5 groups of parameters: in the first 4 groups triplets of parameters show large collinearity, while in the fifth group 5 parameters are collinear

With this aim, we display the model structure in the natural network visualization technique. An example is shown in Fig. 1 c. The model structure is represented as a graph whose nodes are state variables, observables, stimuli, and model parameters. The edges – which can be directed (arrows) or undirected – have the following meaning: an arrow from node A to node B indicates that node B appears in the equation of A. For example, if the dynamic equation of a state *x*_1_ is $\dot x_{1} = p_{1}\cdot x_{2}$, the corresponding graph would show two arrows *x*_2_→*x*_1_ and *p*_1_→*x*_1_.

More formally, we determine how the state, input variables, stimuli and parameters are connected and influence each other through symbolic manipulation of the model Eq. (1). For this purpose we compute: (i) the Jacobian matrix with respect to the states: $J_{i,j}^{ss} =\frac {\partial f_{i}}{\partial x_{j}}$, (ii) the Jacobian of the observation functions with respect to the states: $J_{i,j}^{so} =\frac {\partial g_{i}}{\partial x_{j}}$, (iii) the Jacobian of the systems dynamics with respect to the stimuli $J_{i,j}^{si} =\frac {\partial f_{i}}{\partial u_{j}}$, and (iv) the Jacobian with respect to the parameters $J_{i,j}^{sp} =\frac {\partial f_{i}}{\partial \theta _{j}}$. All these matrices are evaluated symbolically, and then the expressions are converted to a logical 1 (if the symbolic expression is non zero) or 0 when the symbolic result is zero.

Additionally, we can connect parameters by undirected edges if their collinearity is larger than the collinearity threshold.

### Implementation: the VisId software tool

We implemented the techniques proposed in subsections “Practical identifiability analysis” and “Visualization of identifiable subsets” as a MATLAB software package called VisId, which is provided as Additional file 1 and can also be downloaded from GitHub (https://github.com/gabora/visid). It is free software, made available under the terms of the GNU General Public License version 3. The VisId toolbox relies on three other MATLAB toolboxes, which are also freely available: AMIGO2 [59] (https://sites.google.com/site/amigo2toolbox/download), which is used to to store, simulate and calibrate the models; MEIGO [56] (http://www.iim.csic.es/~gingproc/meigo.html), which implements the Variable Neighbouring Search (VNS) optimization method; and (optionally) RRQR (https://www.mpi-magdeburg.mpg.de/1094756/rrqr), which performs the rank revealing QR decomposition used to initialize the global optimizer. Network visualization is performed with Cytoscape [60] (http://www.cytoscape.org/). Further details can be found in Section 4 of Additional file 2.

## Results

In this section we demonstrate the application of the methodology presented in the previous section using several dynamic systems biology models of different type and complexity. Their main characteristics are given in Table 1. First we present detailed results of identifiability analysis and visualization for a model of the TGF- *β* signalling pathway. We also provide similar results for the genetic network that controls the circadian clock in *Arabidopsis thaliana*. Due to their complexity and yet relatively moderate size, these models are well suited as case studies for illustrating the identifiability methodology in depth.

**Table 1:** List of models used as case studies and their characteristics

	TGF- *β*	Circadian	B2	B4
Description	TGF- *β* signaling	Gene network,	Central Carbon	Metabolic model,
	pathway	*A. thaliana*	Metabolism, *E. coli*	Chinese Hamster Ovary
Reference	[61]	[62]	[23, 63]	[23, 64]
Parameters	18	27	116	117
States	21	7	18	34
Outputs	16	2	9	13

Then we study two large scale benchmark problems included in the BioPreDyn-bench collection [23]. Since the analysis of these latter models is more challenging due to their larger size, we start by demonstrating the performance improvements that can be achieved during parameter estimation using the model calibration procedure proposed in Section “Parameter estimation with regularization and global optimization”. Then we perform identifiability analysis and report the corresponding results, including the graphical representation of the identifiable subset using the natural network visualization.

### TGF- *β* signalling pathway

The dynamic model of the TGF- *β* signaling pathway was presented in [61] as a tutorial example for model calibration. It has 18 dynamic states and 21 kinetic parameters (*k*_1_– *k*_21_), of which 18 need to be estimated. Following [61], we assumed that all the concentrations, except the Smad RNAs ($C_{\mathrm {I\_Smad\_mRNA1}}$ and $C_{\mathrm {I\_Smad\_mRNA2}}$), can be measured in the experiments. The algebraic Equations of the reaction kinetics and the dynamic equations are provided in the Additional file 2.

For the purpose of testing the methodology we generated a training dataset by simulating the model equations using the nominal values of the parameters *k*_1_– *k*_21_ (numerical values are listed in Additional file 2: Table S1). Then we sampled the simulated trajectories at equidistant time points, and added normally distributed random numbers to the data to mimic measurement errors. Finally, we estimated the model parameters from the generated data set. This approach is widely used for testing calibration methods and assessing the extent to which they recover the nominal parameters. It should be noted that, as the amount of noise in the dataset increases, the information/signal ratio decreases, making the estimation problem more ill-conditioned. This makes it more difficult to recover the correct value of the parameters, but has a small effect in computation times. The numerical values of the estimated parameters are reported in Additional file 2: Table S2.

We started the identifiability analysis by computing the sensitivities of the observations with respect to the estimated model parameters, according to Algorithm 1. We found that all the parameters have a non-negligible influence on the model outputs, thus there are no individually non-identifiable parameters (see Fig. 1 a).

Next, following Algorithm 2, we applied QR decomposition and ranked the parameters according to their orthogonality. We then solved the optimization problem (23)–(27) by initializing the variable neighboring search method with the results of the QR decomposition (Algorithm 3). Setting the threshold level for the collinearity index to CI =20 yielded 14 identifiable parameters, which are shown as green nodes in the network in Fig. 1 c. Parameters not present in the identifiable subset are shown as red nodes. Parameters are connected by arrows to state variables (represented by yellow nodes) if they appear in the equation of the corresponding dynamic equation. States which directly influence each other are also connected by directed edges in the same manner. Blue squares represent measurements; a state is connected to a blue square if it appears in the corresponding observation function.

To see how the size of the identifiable subset is influenced by the choice of the collinearity index threshold (CI), we solved the optimization problem for a range of threshold values. The results are depicted in Fig. 1 b. As the collinearity index threshold decreases, less parameters are considered identifiable. We can see that the identifiability results are quite robust to the choice of threshold level: the number of identifiable parameters is always between 12 and 15, and it is constant (=14) for a very wide range of CI, 15≤CI≤25.

The results presented so far tell us that the 14 parameters are not correlated. However, they do not inform of the relationships among identifiable and non-identifiable parameters. To investigate this point, we computed the smallest correlated subsets as described in Section “Partitioning the non-identifiable parameters”, up to groups of 6 parameters. Figure 1 d shows such groups; parameters are depicted as blue circles connected with group identifying nodes (white squares). These nodes are labeled as GX(Y), where X indicates the number of parameters in the group and Y is the group index for a given number of parameters (e.g. G3(2) stands for the second group of three correlated parameters). We found that the large pairwise collinearity between *k*_14_−*k*_18_ and *k*_17_−*k*_19_ explains the non-identifiability of the model parameters only partially. There are 4 groups of triplets and a group of 5 parameters which are highly correlated. The members of the groups and the corresponding collinearity index are reported in Table 2.

**Table 2:** TGF- *β* model: highly collinear parameter sets. A set ID indicates the number of parameters involved in the collinearity group. They are also depicted in Fig. 1 d

Set ID.	CI	Parameters				
G2(1)	2.87e+07	k14	k18			
G2(2)	41.4	k17	k19			
G3(1)	110	k14	k16	k19		
G3(2)	1.37e+03	k14	k16	k17		
G3(3)	1.37e+03	k16	k17	k18		
G3(4)	110	k16	k18	k19		
G5(1)	22.9	k8	k9	k10	k11	k12

It is important to note that collinearity might arise among multiple parameters, even if they are pairwise independent. For example, despite the fact that none of the pairs in the group of *k*_14_, *k*_16_ and *k*_17_ has a high pairwise collinearity, the collinearity index of the triplet is extremely large.

Algorithm 4 found 40 different sets of identifiable parameters with collinearity index ranging between 12.4 and 16, less than the threshold (CI = 20). The sets are reported with the corresponding collinearity index in Additional file 2: Table S3. We can see that parameters {*k*_1_−*k*_7_, *k*_13_, *k*_15_} are members of all the groups, and they do not participate in any of the small correlated groups in Fig. 1 d. From each correlated group of size *K*, only *K*−1 parameters can participate in the largest set of identifiable parameters.

The aforementioned identifiability procedures can be carried out in a few seconds. Detailed computational costs are shown in Table S6 of the Additional file 2 for all the case studies considered in this paper.

### Circadian clock in *Arabidopsis thaliana*

Locke and co-authors [62] described the genetic network controlling the circadian clock in *Arabidopsis thaliana*; the dynamic equations of this model are provided in the Additional file 2.

We generated training data by simulating the model equations with the nominal parameters (Additional file 2: Table S4) in two experimental conditions. In the first one, the model input was kept constant (*θ*_light_=1), representing continuous light stimulation of the plant. In the second experiment the input was changed pulse-wise in 12-hour cycles, repeated 5 times. As in the previous example, the trajectories were sampled at equidistant time-points and disturbed by pseudo-random noise. Only two states, *C* *T*_*m*_ and *C* *L*_*m*_, were observed. The estimated model parameters are collected in Additional file 2: Table S4.

**Fig. 2:**
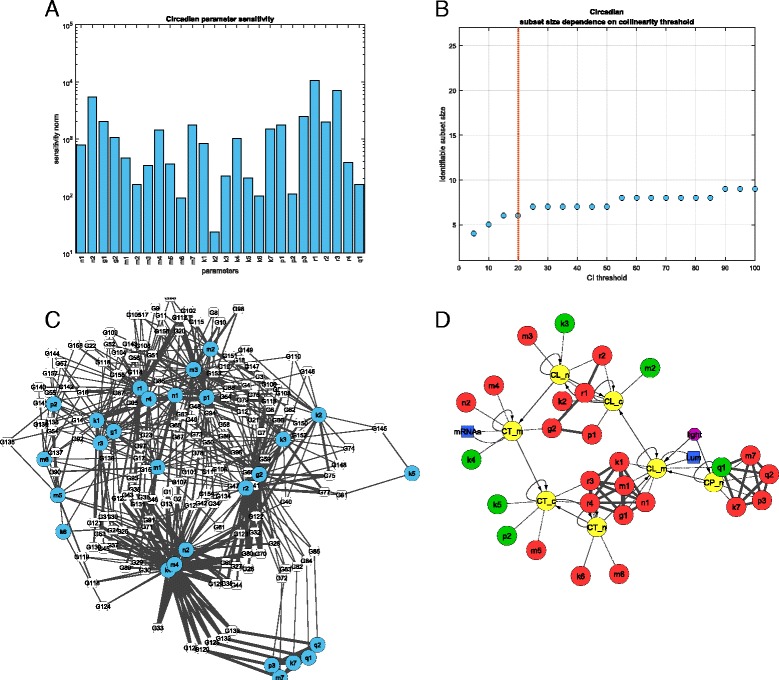
Circadian clock in *Arabidopsis thaliana*: panel **a** shows the model sensitivities with respect to parameters in logarithmic scale. In panel **b** the maximum number of identifiable parameters is depicted as a function of the collinearity threshold. The *red vertical line* indicates our choice of threshold (CI =20) used for further analysis. In panel **c** the interplay among the collinear parameters is indicated up to groups of 3 parameters. In panel **d** the mathematical model is depicted. Nodes indicate states (*yellow*), identifiable (*green*) and not identifiable (*red*) parameters, and observables (*blue*). Highly correlated parameter pairs are connected by undirected edges

Although the model outputs showed sensitivity to all the parameters (Fig. 2 a), i.e. there were no zero sensitivity vectors, we found that most of the model parameters are non-identifiable due to heavy collinearities. The largest identifiable subset contains only 6 of the 27 parameters, depicted in Fig. 2 d by green nodes. The enumeration of the largest sets of identifiable parameters by Algorithm 4 identified 1331 parameter sets.

### Benchmarks B2 and B4 from the BioPreDyn-bench collection

In this subsection we analyze two large scale benchmark problems taken from the BioPreDyn-bench collection [23]: the metabolic models of *Escherichia coli* (B2) and Chinese Hamster Ovary cells (B4). They are highly non-linear, partially observed systems with more than 100 unknown parameters, which pose serious challenges for parameter identification. In B4 the calibration data was generated by model simulation and disturbed by random noise, while in B2 it was experimentally measured. Further details about the models and the parameter estimation challenge can be found in [23].

First we use these benchmarks to illustrate the benefits of the parameter estimation strategy proposed in subsection “Parameter estimation with regularization and global optimization”, comparing it with the one used in [23]. Both approaches use a hybrid method, eSS [21], which combines a global optimization algorithm (scatter search) with a local search. In [23] the local method of choice was FMINCON; here we compare that configuration with NL2SOL (with and without regularization). Global optimization algorithms use pseudo-random numbers. Hence their performance changes at every run, and the calibration problem should be solved several times to obtain more robust results. Since each optimization takes several hours we limited the number of runs to five for each problem. We used the approximate computation time (CPU time) reported in [23] as the stopping criterion for the model calibration. Convergence curves depict the best objective function value found versus CPU time, and can be used to compare the performance of different algorithms. An optimization method is preferred if it achieves a lower objective function value at earlier CPU time. The best convergence curves (out of 5) corresponding to B2 and B4 are shown in the Additional file 2 for 3 algorithms: (1) eSS-FMINCON, as reported in [23]; (2) eSS-NL2SOL; and (3) eSS-NL2SOL using regularization as recommended in Section “Parameter estimation with regularization and global optimization”. From those curves we see that the algorithm (3) proposed here converged earlier than the others to the optimal objective function value (note that log-log scale is used in these curves). We stress that the main purpose of regularization is to avoid overfitting: we do not wish to obtain an excessively good fit, which would indicate that we are reproducing noise instead of the true dynamics. Therefore, regularization should *not* achieve a smaller objective function value.

**Fig. 3:**
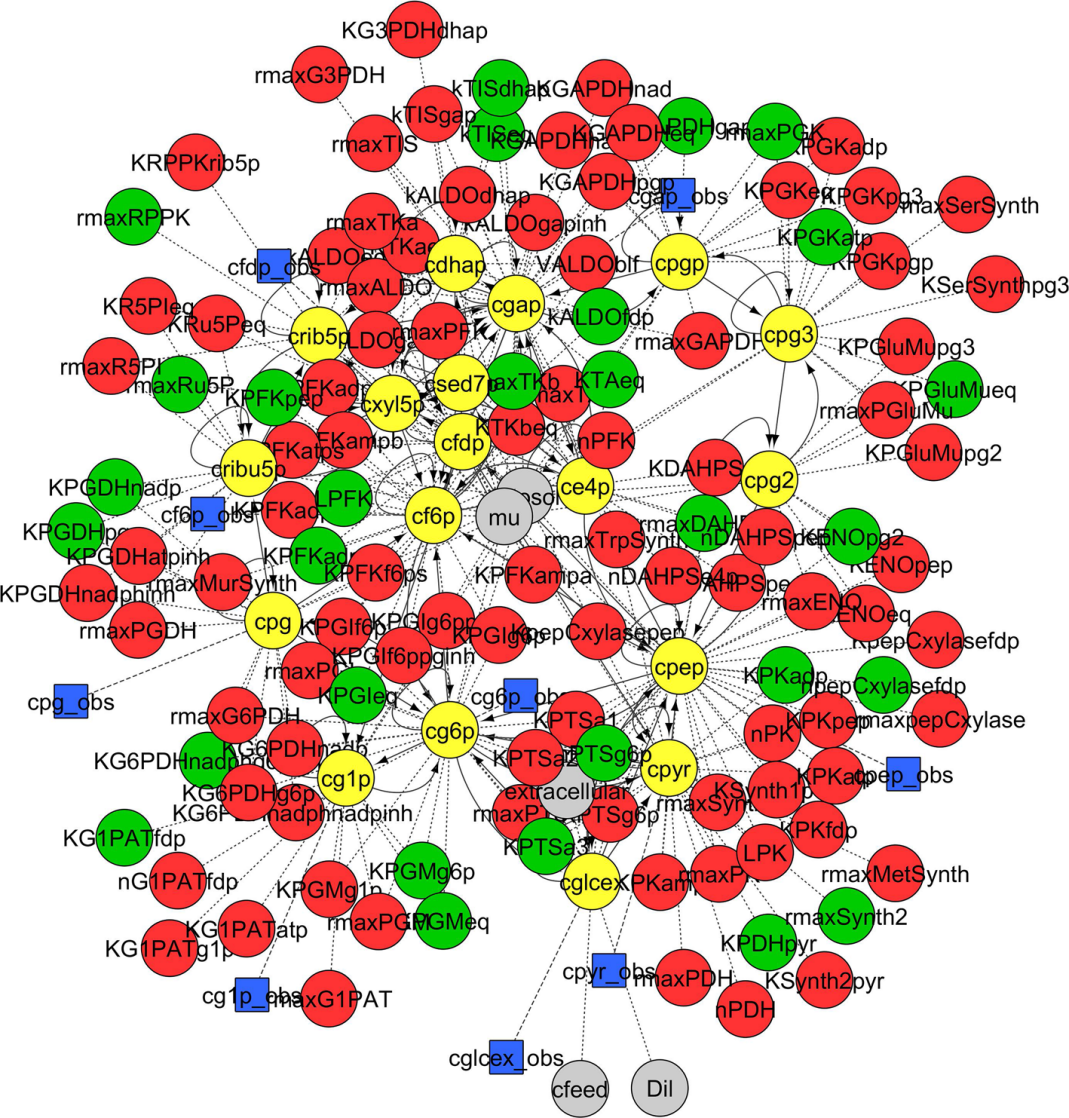
Representation of the connections in the B2 model using the network diagram formalism. Nodes indicate states (*yellow*), identifiable (*green*) and not identifiable (*red*) parameters, observables (*blue*), and inputs (*grey*). The source file of this figure is provided with the VisId toolbox; using Cytoscape, the user can navigate through it and zoom on different areas to improve the visibility

**Fig. 4:**
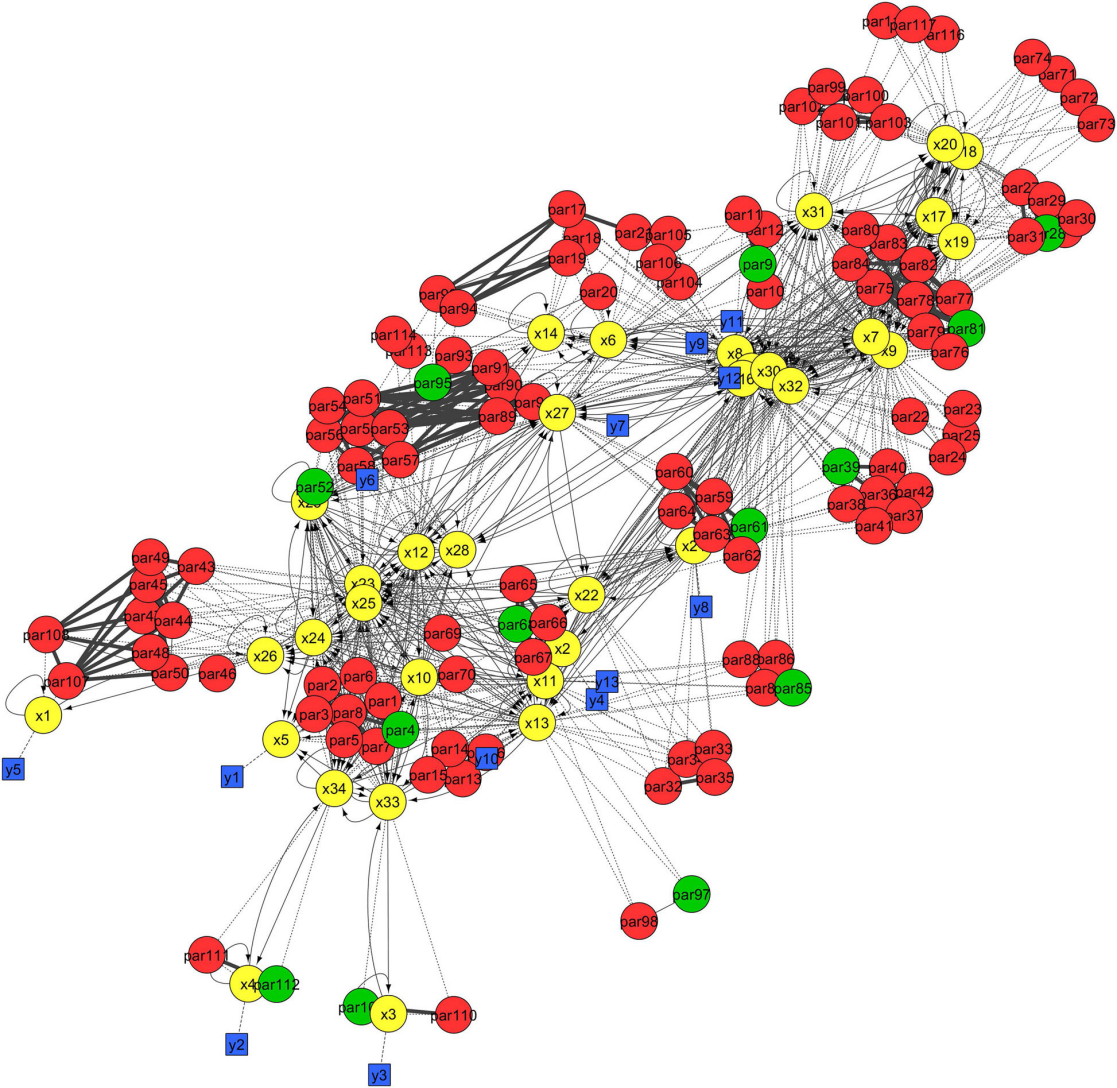
Representation of the connections in the B4 model using the network diagram formalism. Nodes indicate states (*yellow*), identifiable (*green*) and not identifiable (*red*) parameters, observables (*blue*), and inputs (*grey*)

**Fig. 5:**
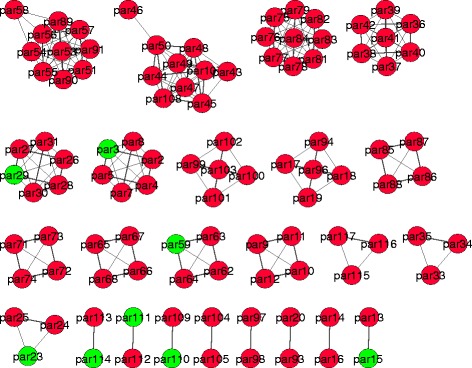
Visualization of the relationships among highly collinear parameters in the B4 model. The figure shows small groups, whose sizes range between 2 and 10 parameters. Unidentifiable parameters are shown in *red*; identifiable parameters in *green*. Highly correlated pairs are connected by lines

Next, we apply the identifiability analysis procedures presented in subsections “Practical identifiability analysis” and “Visualization of identifiable subsets” to these two models. For B2 they yield an identifiable subset of size 29, and for B4 of size 13 (recall that both models have a total of 116 parameters). The corresponding networks are shown in Figs. 3 and 4, respectively. It is also possible to find small groups of highly correlated parameters for models of this size; e.g. for B4 we obtained those depicted in Fig. 5.

The aforementioned results show that both models are poorly identifiable in practice for the considered datasets; more informative data would be needed in order to obtain accurate estimates of their parameters.

## Discussion and conclusions

In this paper we have presented a workflow to efficiently estimate the parameters of dynamic models and analyze their practical identifiability. Our approach combines an advanced optimization technique, which reduces computation times in parameter estimation, and several identifiability analysis procedures, which can find subsets of identifiable and unidentifiable parameters. Results are visualized using network diagrams, which provide an intuitive representation of the findings and facilitate their analysis and understanding.

Many approaches have been applied to study identifiability of kinetic models, but they suffer from lack of scalability. An advantage of the integrated method presented here is its moderate computational cost, which enables its application to large-scale models; complete results can be obtained in a few hours for models of more than a hundred parameters. Another important aspect is the integration of identifiability analysis with visualization, which presents the results in a way that is easily interpretable for modelers and experimentalists. Currently, its main limitation arises when trying to find *all* the different existing groups of highly correlated parameters: the combinatorial explosion of this particular task makes it feasible only for models of moderate size, i.e. of a few dozens of parameters. However, all the remaining steps of the workflow presented in this manuscript scale up well up to several hundred parameters.

The usefulness of the methodology and workflow presented here goes beyond basic parameter identifiability analysis. The procedure not only (i) determines the largest subset of identifiable parameters, but also (ii) informs about the characteristics of the space of non-identifiable parameters, reporting small groups of highly correlated parameters, and (iii) presents all these results in a coherent and scalable way using visualization techniques, facilitating the understanding of the underlying complex interactions. Uncovering these higher order relationships helps in determining the causes of unidentifiability and provides guidelines for remedying them, e.g. by reformulating the model or by collecting new data through a new experimental design. All this information can be readily used to improve the iterative model-building cycle.

A MATLAB implementation of the identifiability and visualization methodology, which we have called the VisId software package (Additional file 1), is available from GitHub (https://github.com/gabora/visid) as free, open source software. This distribution includes the case studies discussed above.

## Additional files

Additional file 1 VisId toolbox. This compressed folder contains the VisId MATLAB toolbox. (ZIP 1030 KB)

Additional file 2 Supplementary material. This document contains detailed descriptions of the case studies and of the VisId toolbox, as well as additional details about the results. (PDF 306 KB)

## Abbreviations

CCA: Canonical correlation analysis
CI: Collinearity index
CPU: Central processing unit
eSS: Enhanced scatter search
MI: Mutual information
ODE: Ordinary differential equation
QR: Decomposition of a matrix into an orthogonal matrix (Q) and an upper triangular matrix (R)
RNA: Ribonucleic acid
RRQR: Rank revealing QR decomposition
TGF- *β*: Transforming growth factor beta
VNS: Variable neighbourhood search
