# Supplementary figures and images for: Parameter identifiability analysis and visualization in large-scale kinetic models of biosystems
Source: BMC Syst Biol. 2017 May 5;11:54. doi: 10.1186/s12918-017-0428-y (PMC5420165; doi:10.1186/s12918-017-0428-y)

**B2**

**subset size dependence on collinearity threshold**

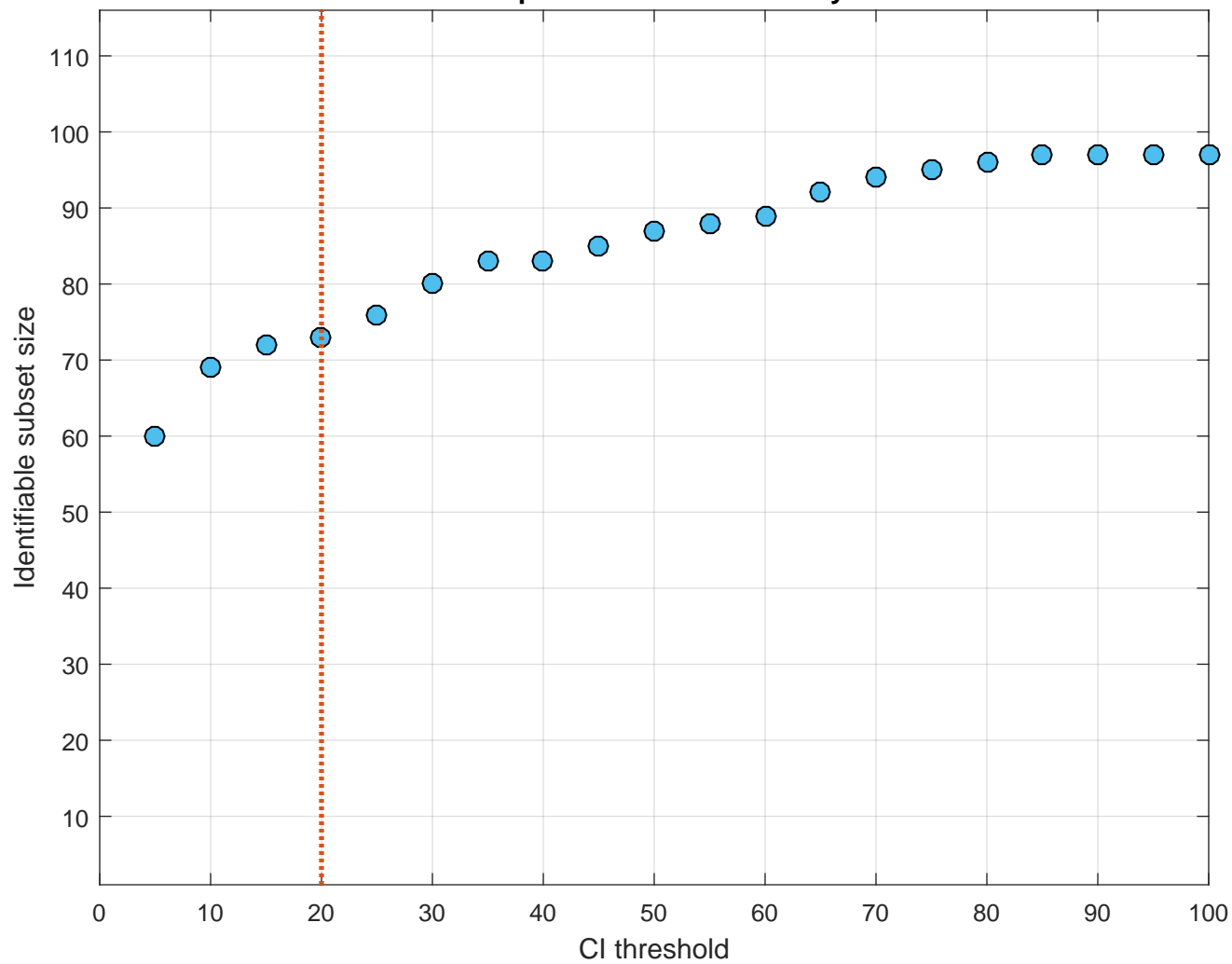

Supplement: Supplementary file 1 — VisId toolbox. This compressed folder contains the VisId MATLAB toolbox. (ZIP 1030 KB) [file 12918_2017_428_MOESM1_ESM.zip › visid-master/case_studies/B2/B2_CI_idsubsetsize.pdf]

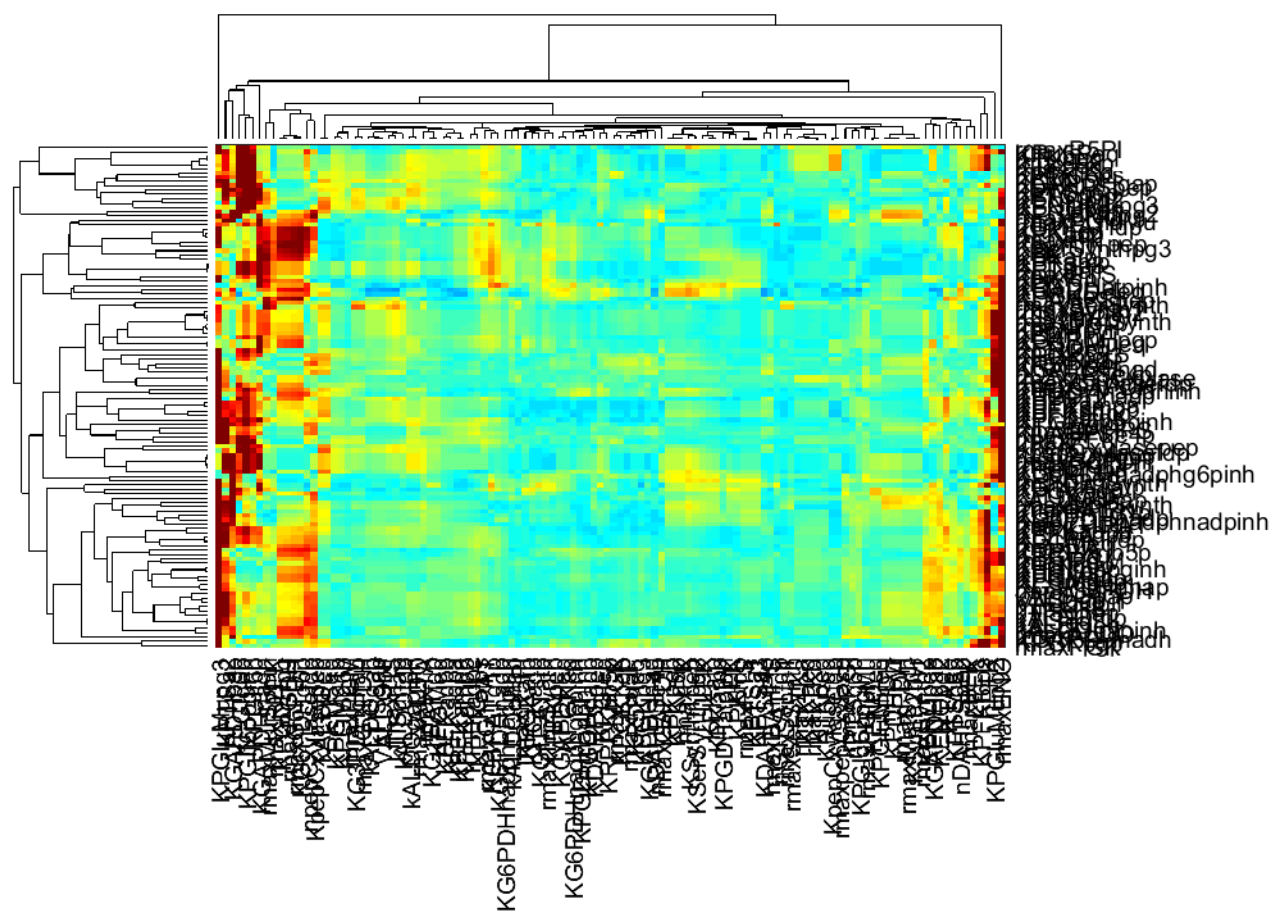

Supplement: Supplementary file 1 — VisId toolbox. This compressed folder contains the VisId MATLAB toolbox. (ZIP 1030 KB) [file 12918_2017_428_MOESM1_ESM.zip › visid-master/case_studies/B2/B2_clustCorrmat.pdf]

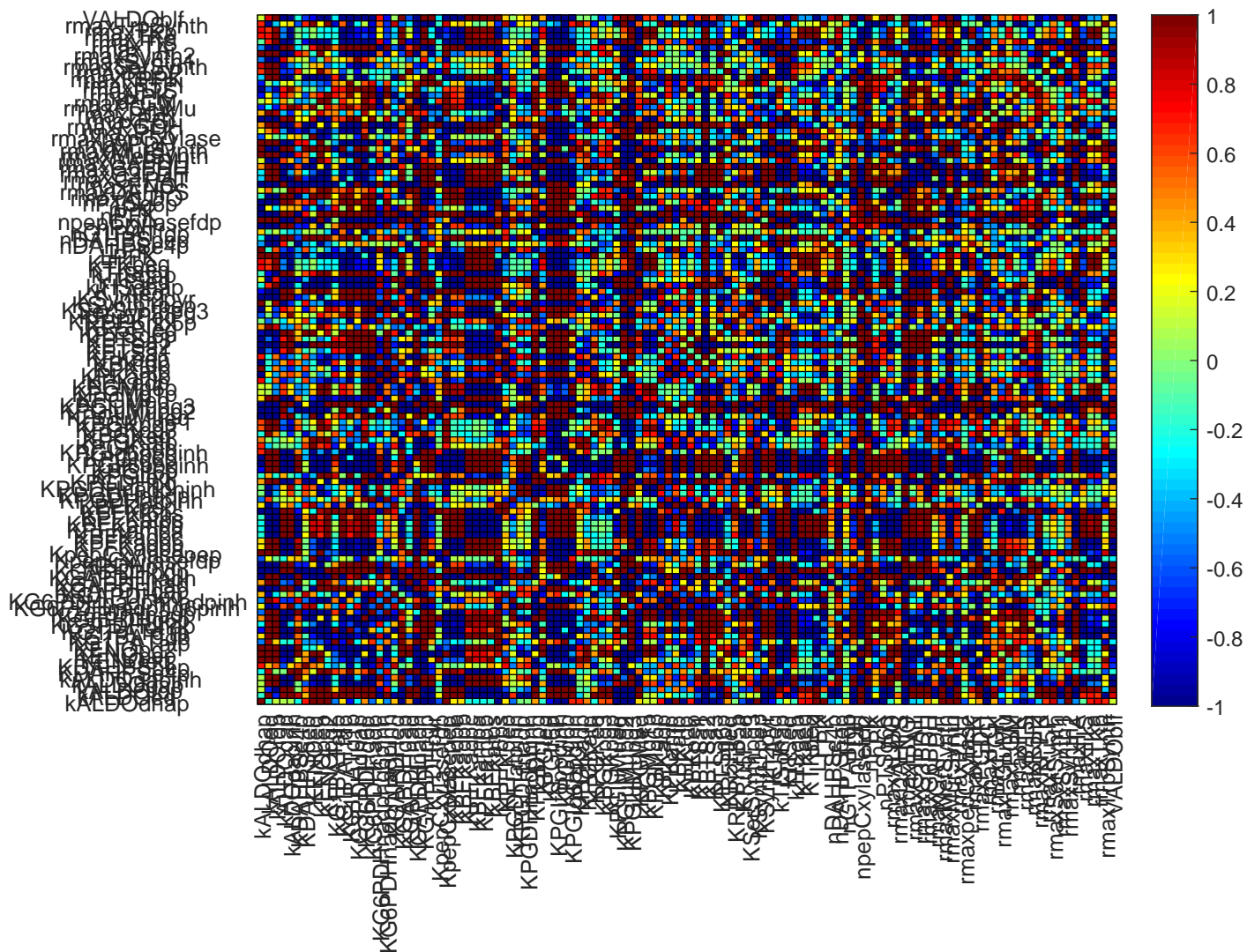

Supplement: Supplementary file 1 — VisId toolbox. This compressed folder contains the VisId MATLAB toolbox. (ZIP 1030 KB) [file 12918_2017_428_MOESM1_ESM.zip › visid-master/case_studies/B2/B2_corrmat.pdf]

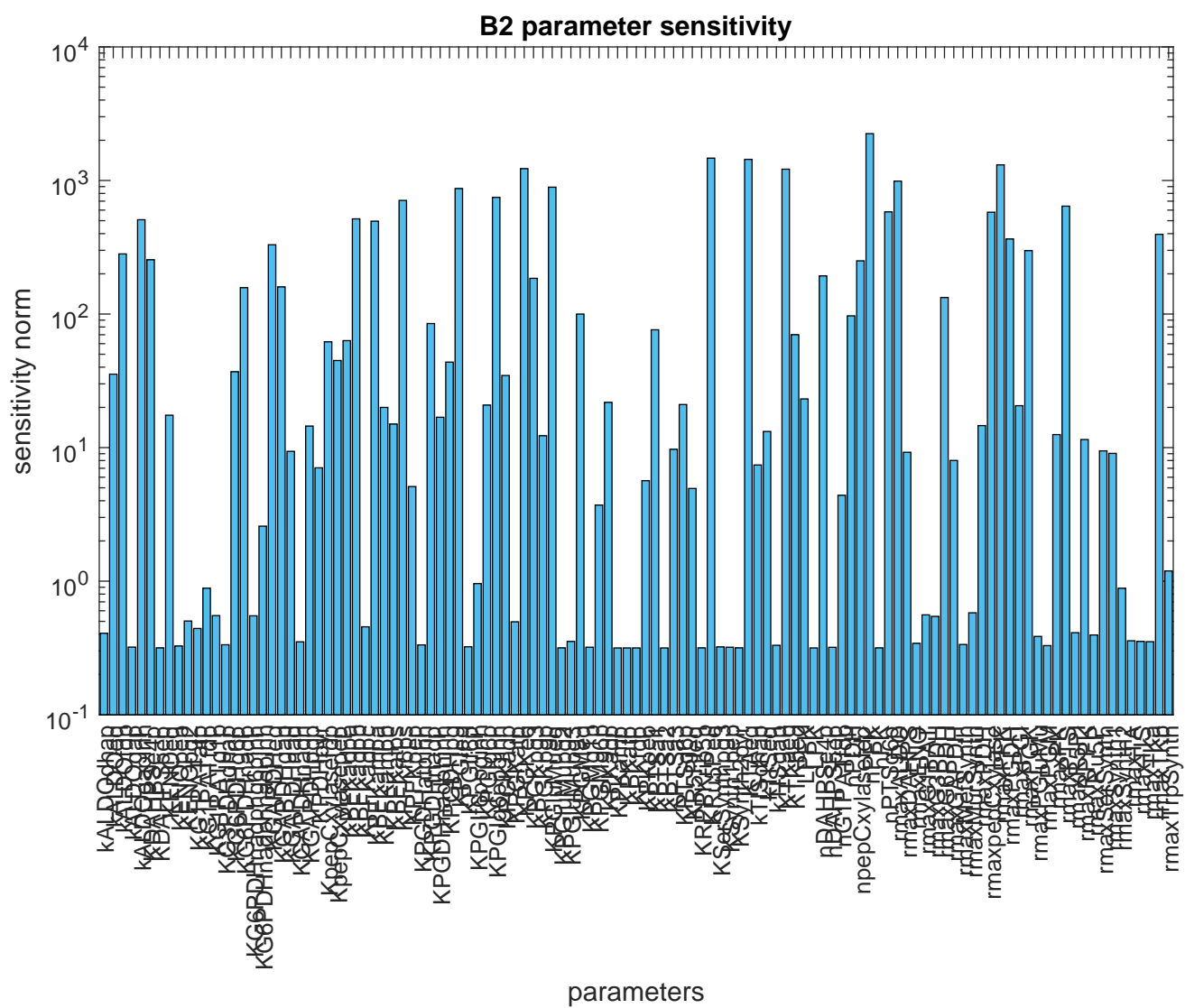

Supplement: Supplementary file 1 — VisId toolbox. This compressed folder contains the VisId MATLAB toolbox. (ZIP 1030 KB) [file 12918_2017_428_MOESM1_ESM.zip › visid-master/case_studies/B2/B2_sensitivity_bar.pdf]

**B4**

**subset size dependence on collinearity threshold**

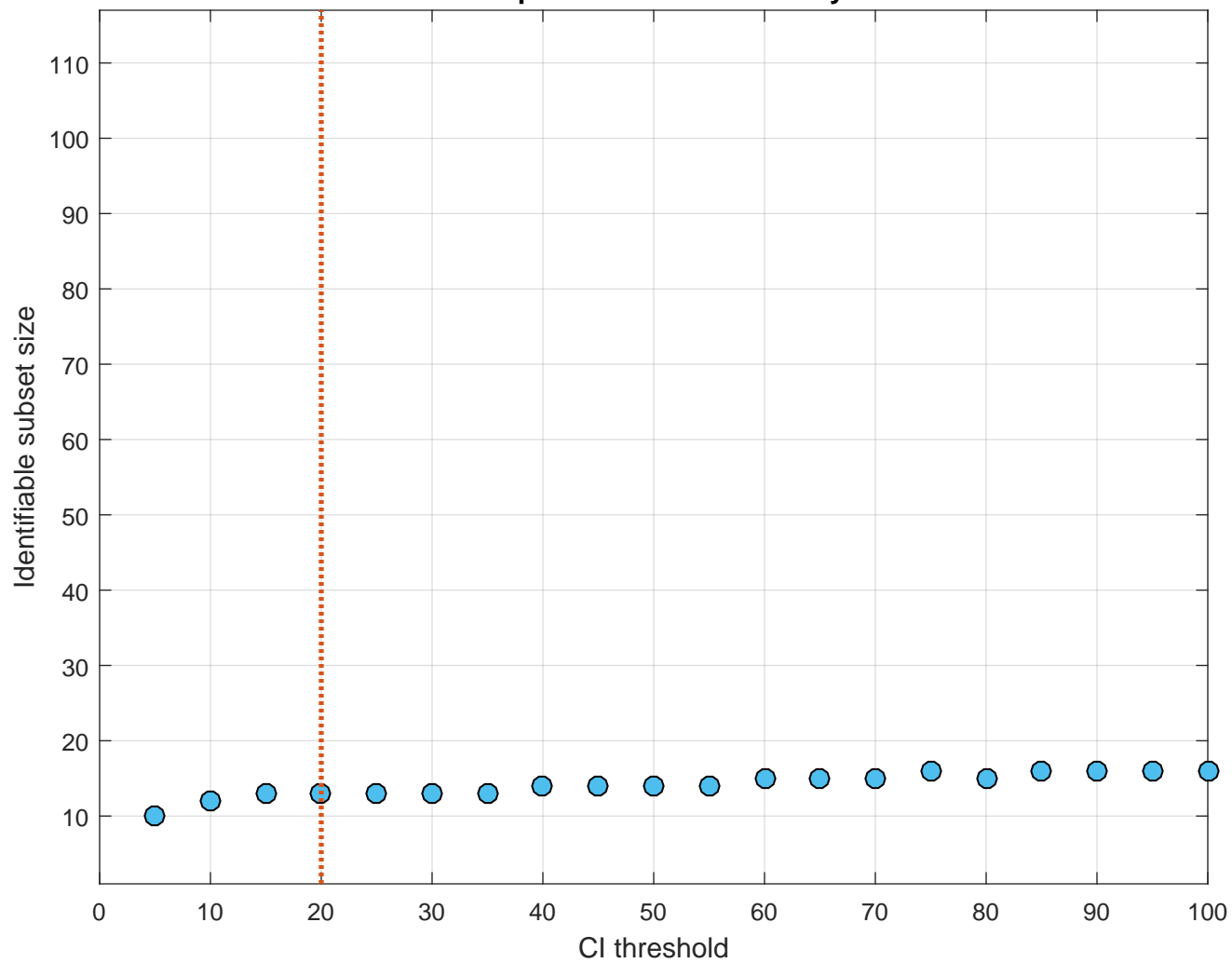

Supplement: Supplementary file 1 — VisId toolbox. This compressed folder contains the VisId MATLAB toolbox. (ZIP 1030 KB) [file 12918_2017_428_MOESM1_ESM.zip › visid-master/case_studies/B4/B4_CI_idsubsetsize.pdf]

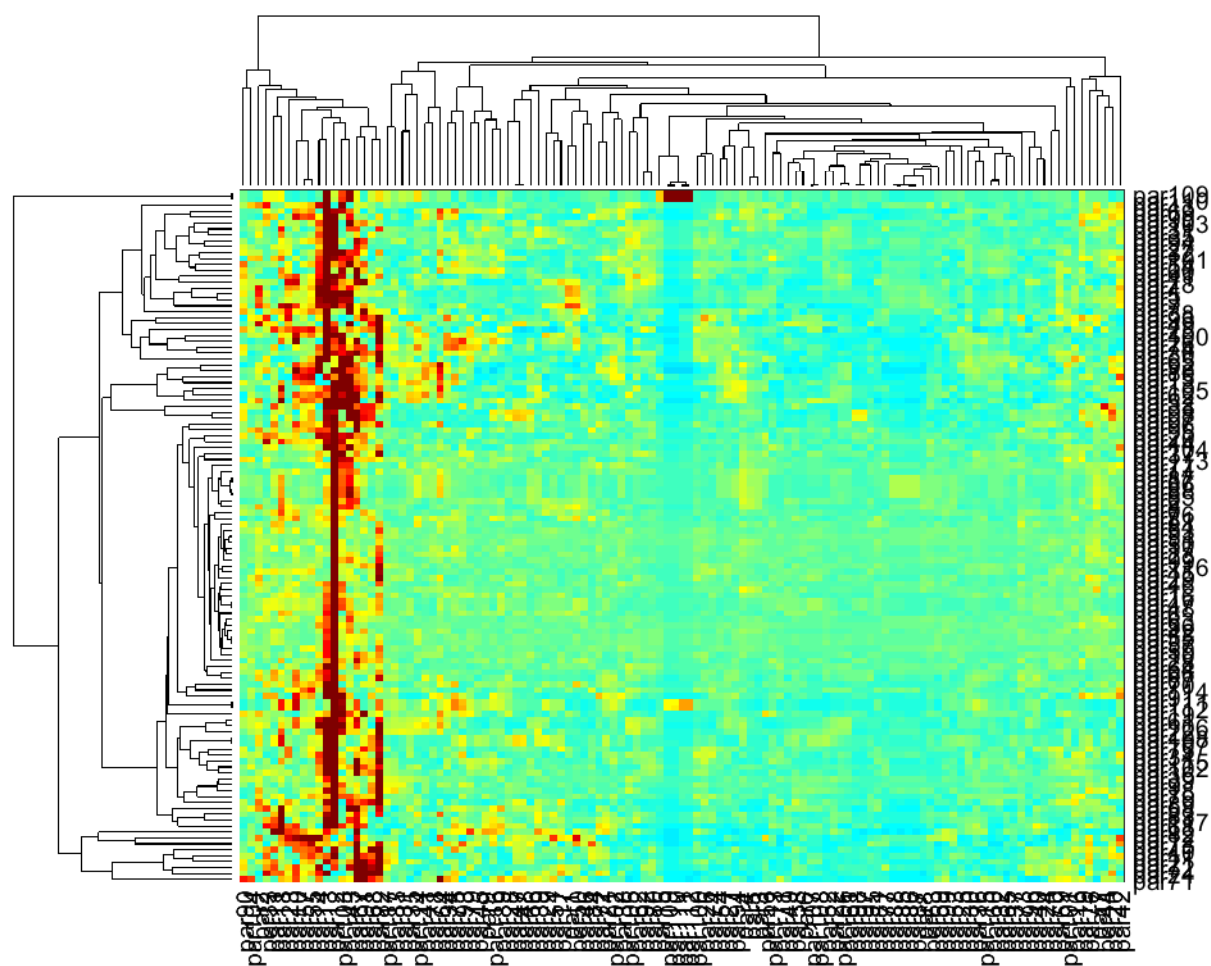

Supplement: Supplementary file 1 — VisId toolbox. This compressed folder contains the VisId MATLAB toolbox. (ZIP 1030 KB) [file 12918_2017_428_MOESM1_ESM.zip › visid-master/case_studies/B4/B4_clustCorrmat.pdf]

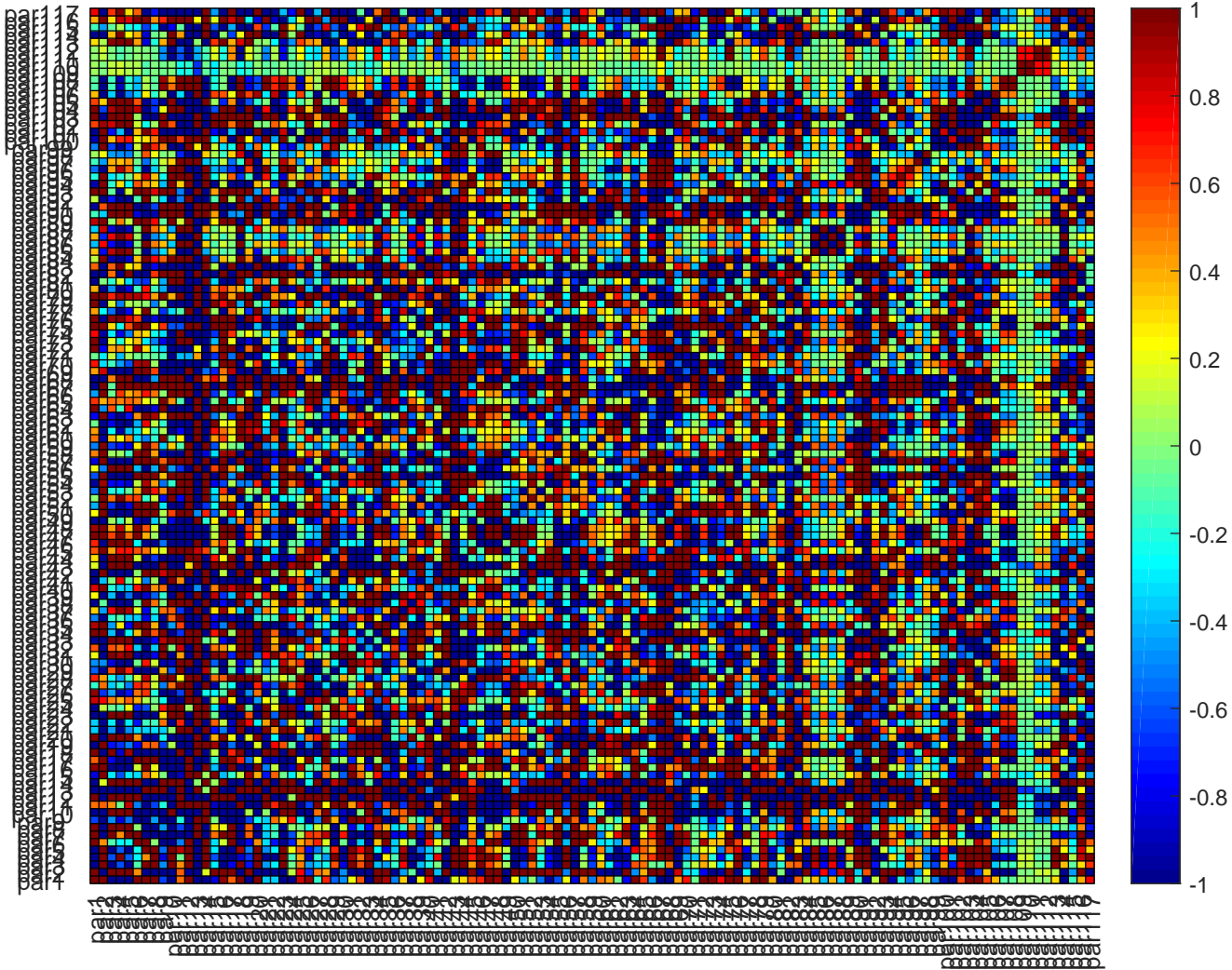

Supplement: Supplementary file 1 — VisId toolbox. This compressed folder contains the VisId MATLAB toolbox. (ZIP 1030 KB) [file 12918_2017_428_MOESM1_ESM.zip › visid-master/case_studies/B4/B4_corrmat.pdf]

**B4 parameter sensitivity**

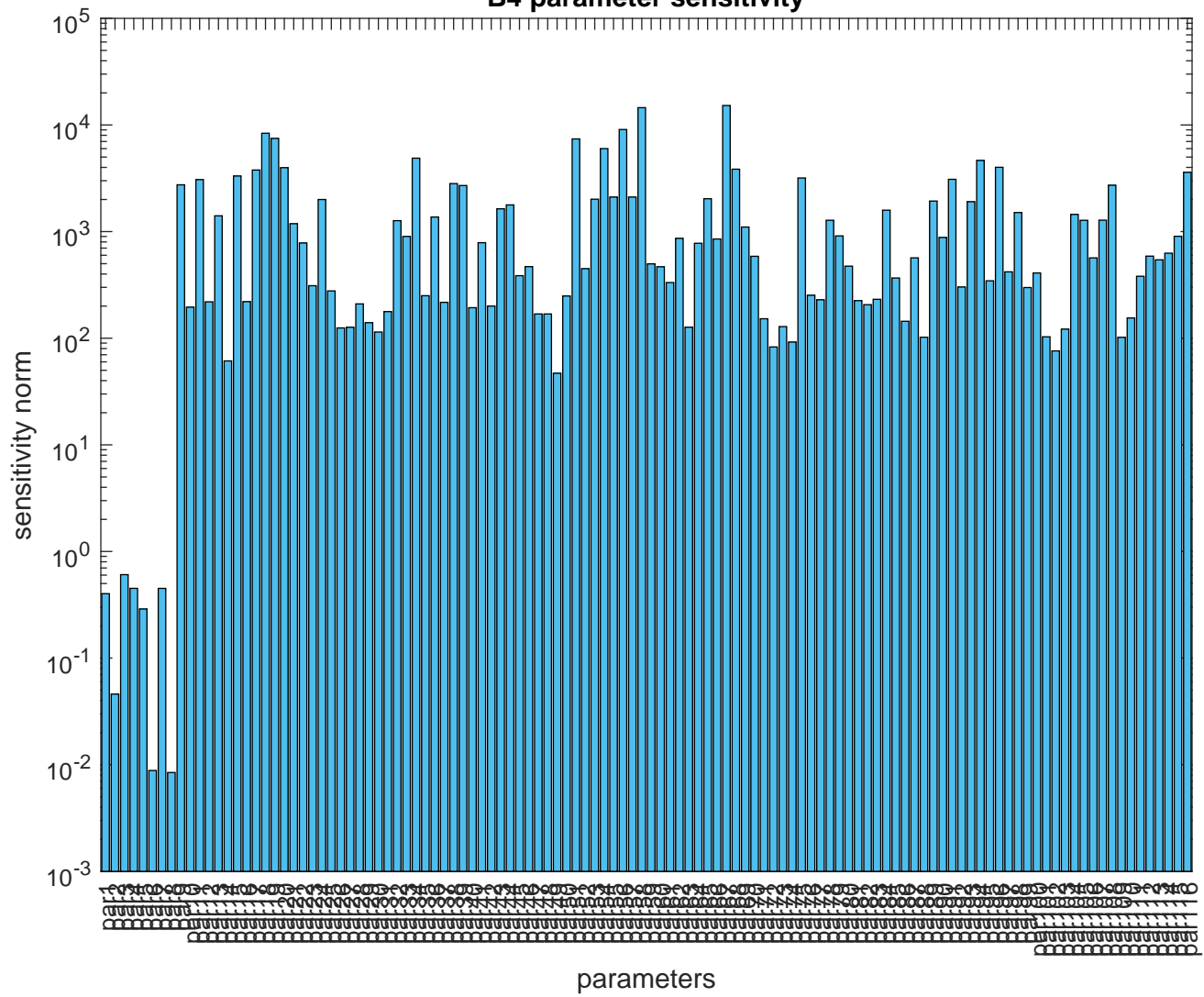

Supplement: Supplementary file 1 — VisId toolbox. This compressed folder contains the VisId MATLAB toolbox. (ZIP 1030 KB) [file 12918_2017_428_MOESM1_ESM.zip › visid-master/case_studies/B4/B4_sensitivity_bar.pdf]

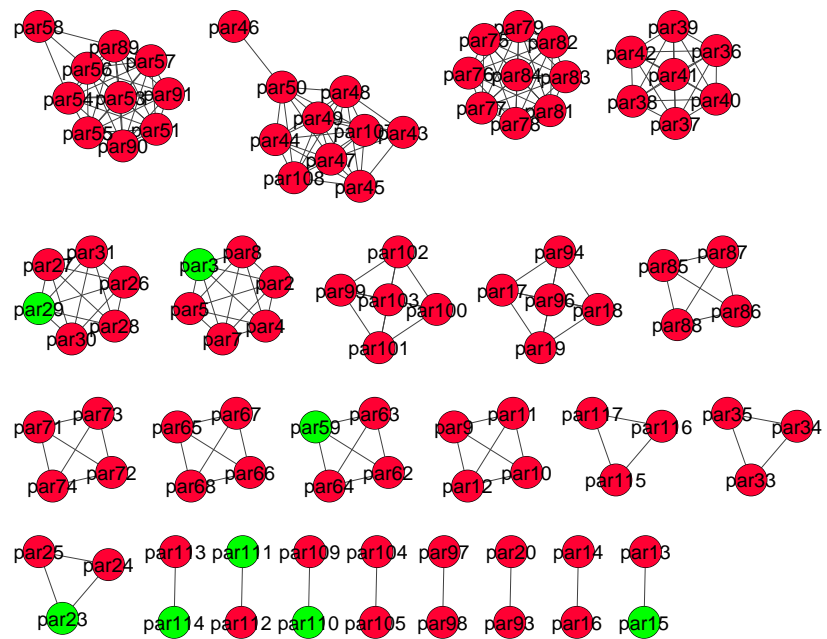

Supplement: Supplementary file 1 — VisId toolbox. This compressed folder contains the VisId MATLAB toolbox. (ZIP 1030 KB) [file 12918_2017_428_MOESM1_ESM.zip › visid-master/case_studies/B4/cytoscape/pairwise_collinearity_groups.pdf]

**Circadian**  
**subset size dependence on collinearity threshold**

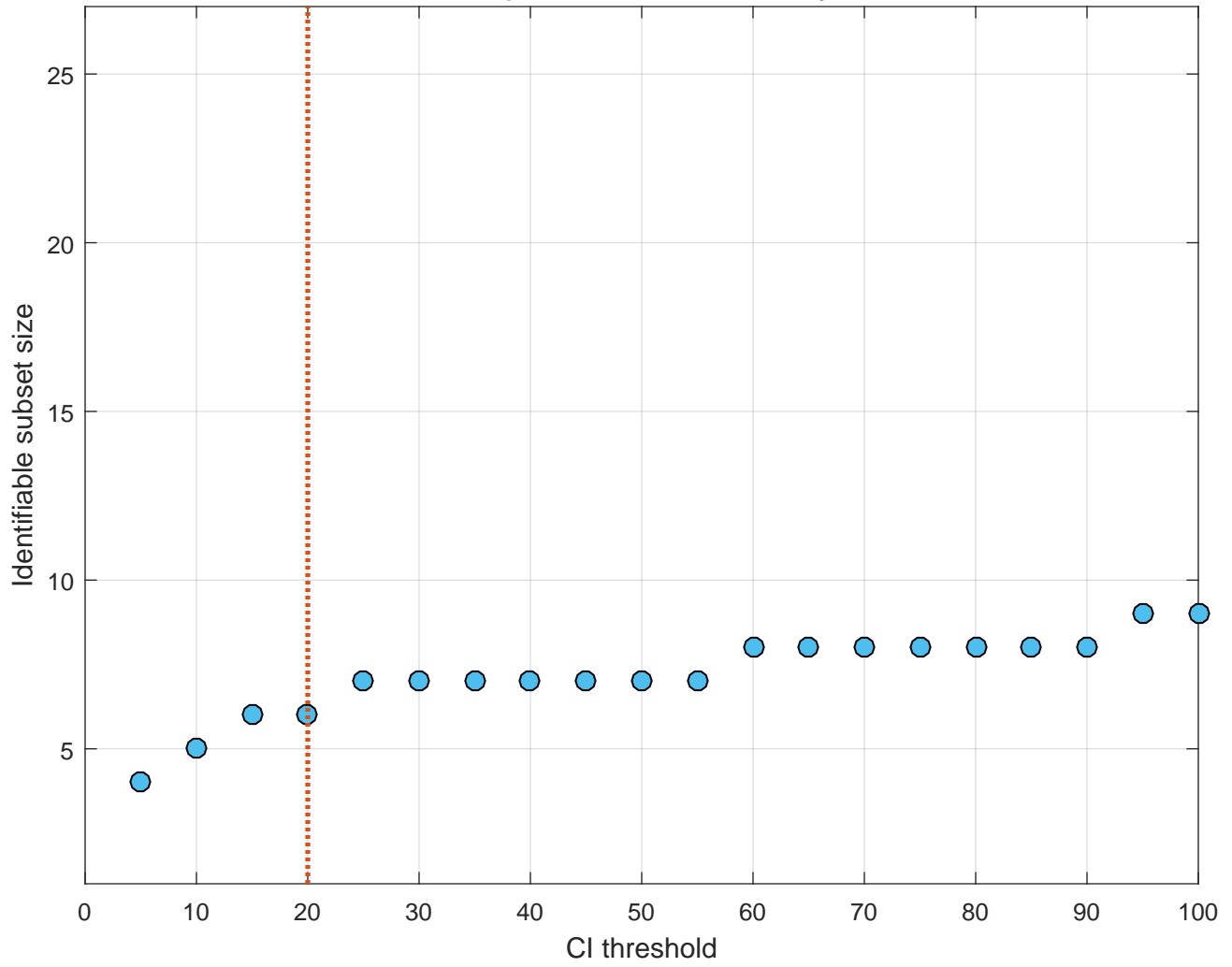

Supplement: Supplementary file 1 — VisId toolbox. This compressed folder contains the VisId MATLAB toolbox. (ZIP 1030 KB) [file 12918_2017_428_MOESM1_ESM.zip › visid-master/case_studies/circadian/circadian_CI_idsubsetsize.pdf]

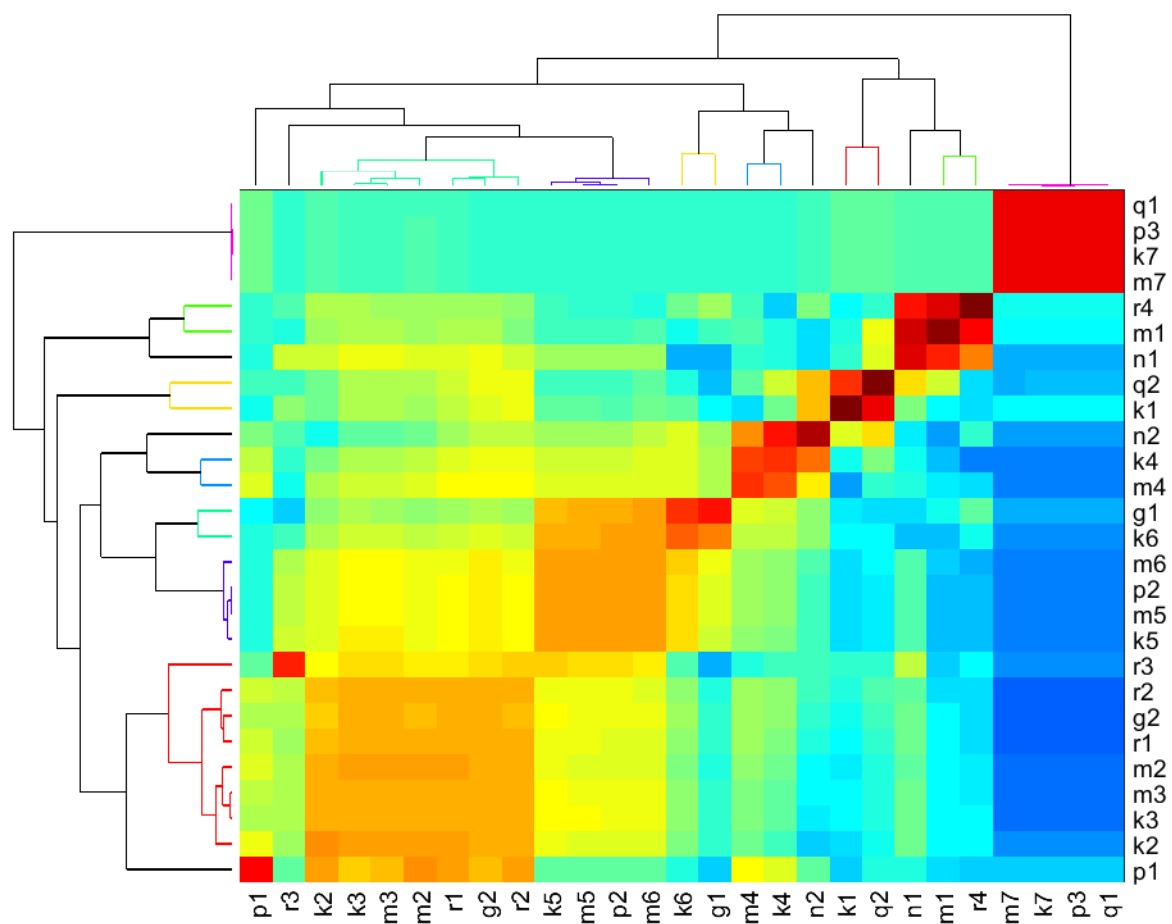

Supplement: Supplementary file 1 — VisId toolbox. This compressed folder contains the VisId MATLAB toolbox. (ZIP 1030 KB) [file 12918_2017_428_MOESM1_ESM.zip › visid-master/case_studies/circadian/circadian_clustCorrmat.pdf]

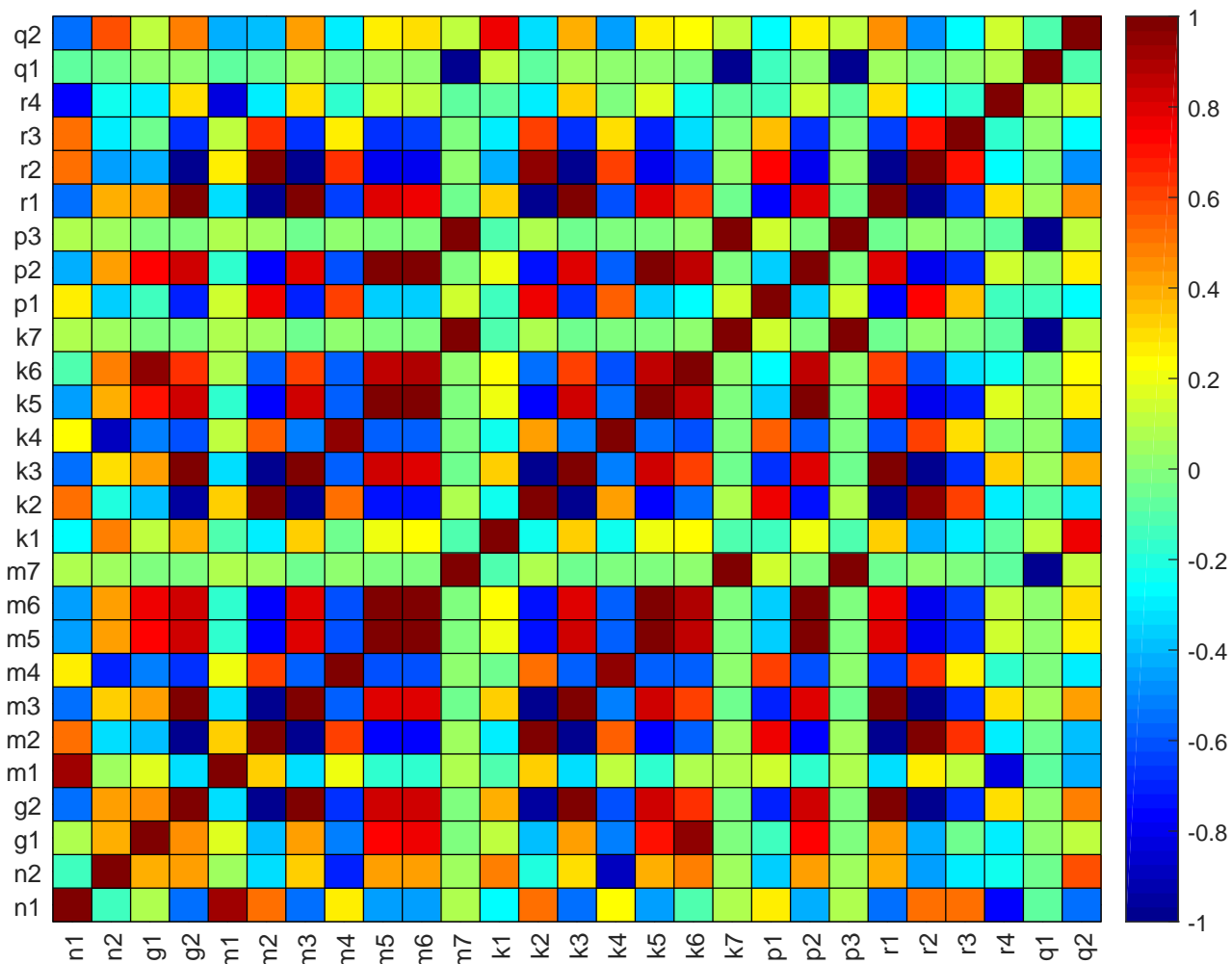

Supplement: Supplementary file 1 — VisId toolbox. This compressed folder contains the VisId MATLAB toolbox. (ZIP 1030 KB) [file 12918_2017_428_MOESM1_ESM.zip › visid-master/case_studies/circadian/circadian_corrmat.pdf]

Circadian parameter sensitivity

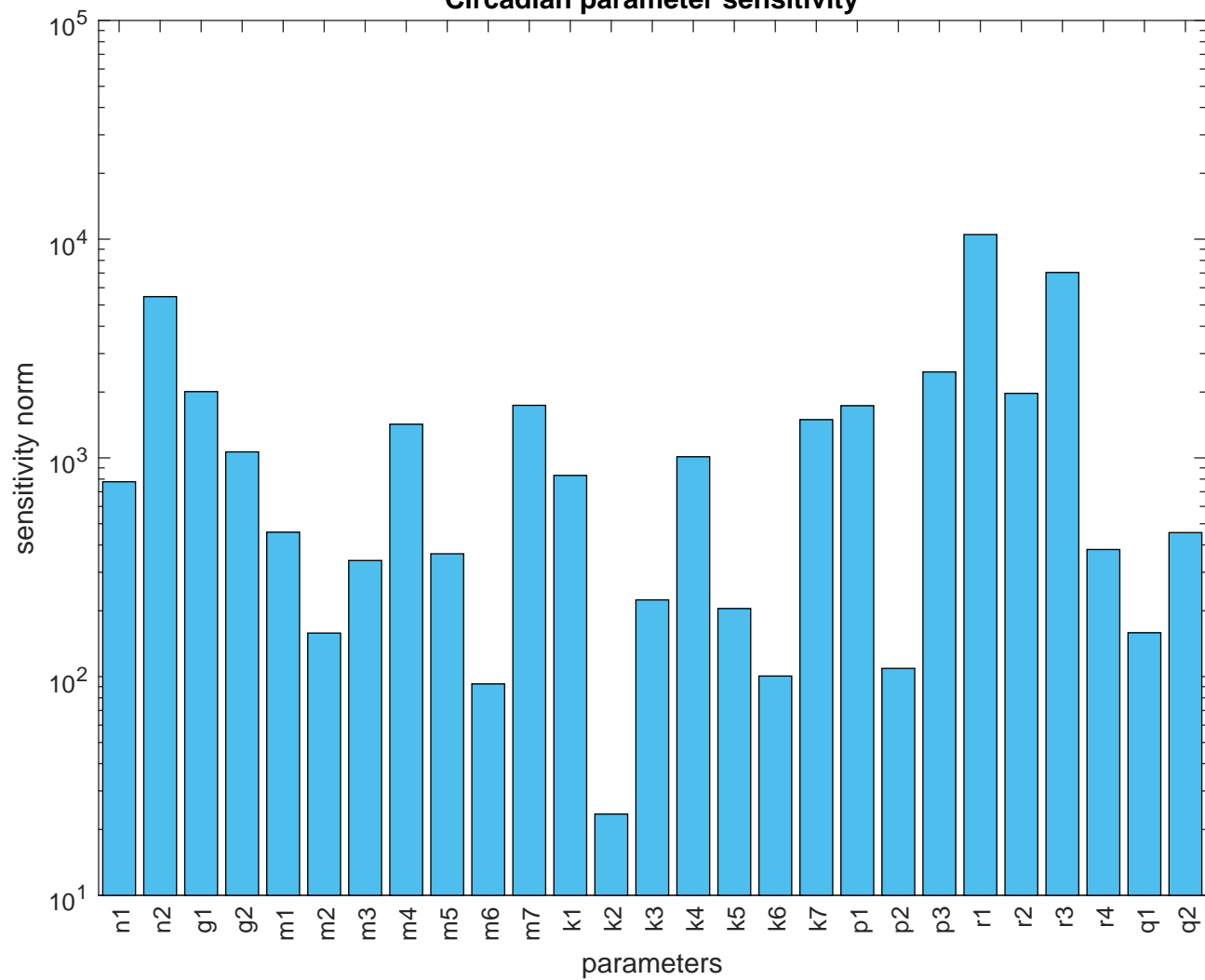

Supplement: Supplementary file 1 — VisId toolbox. This compressed folder contains the VisId MATLAB toolbox. (ZIP 1030 KB) [file 12918_2017_428_MOESM1_ESM.zip › visid-master/case_studies/circadian/circadian_sensitivity_bar.pdf]

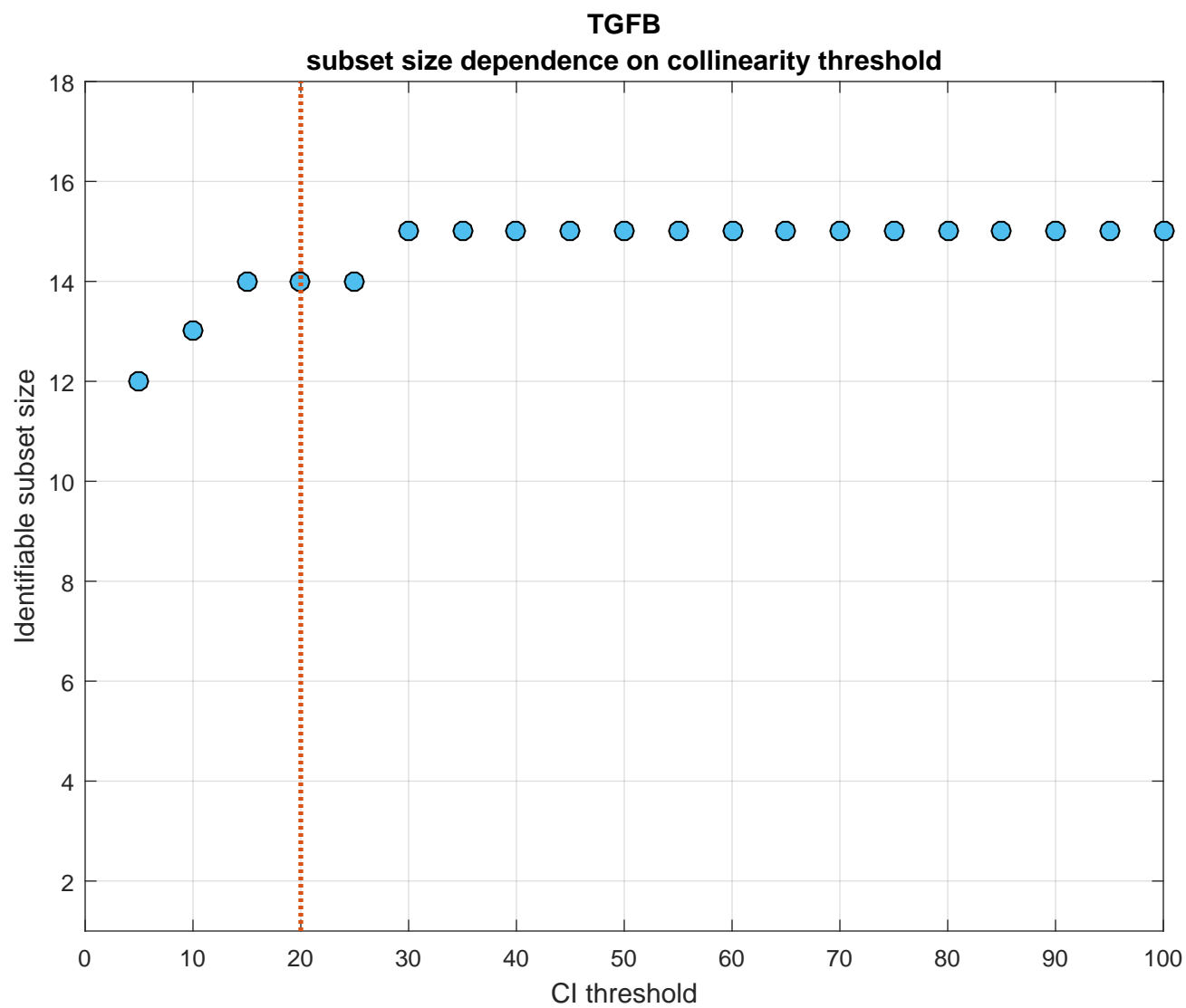

Supplement: Supplementary file 1 — VisId toolbox. This compressed folder contains the VisId MATLAB toolbox. (ZIP 1030 KB) [file 12918_2017_428_MOESM1_ESM.zip › visid-master/case_studies/tgfb/tgfb_CI_idsubsetsize.pdf]

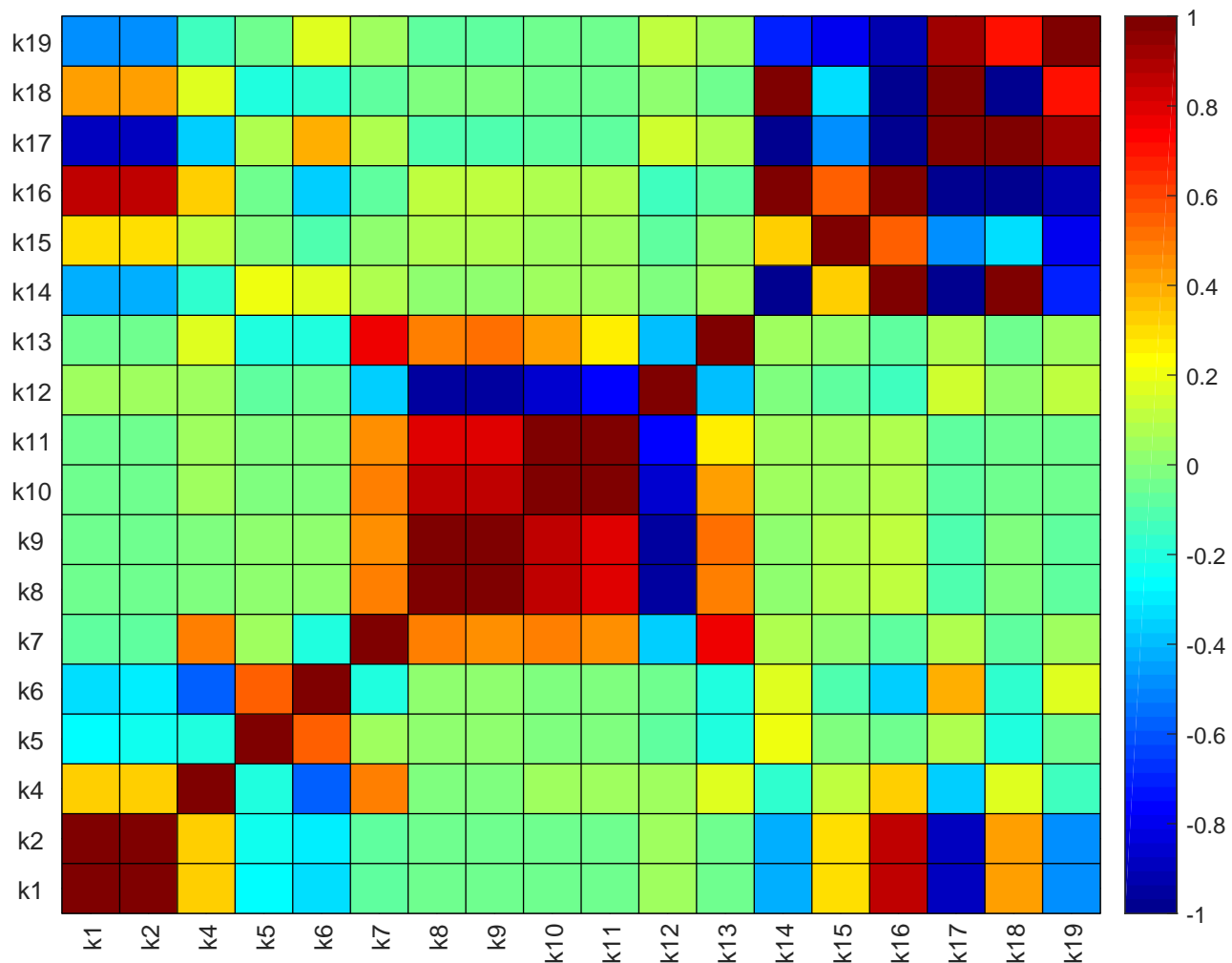

Supplement: Supplementary file 1 — VisId toolbox. This compressed folder contains the VisId MATLAB toolbox. (ZIP 1030 KB) [file 12918_2017_428_MOESM1_ESM.zip › visid-master/case_studies/tgfb/tgfb_corrmat.pdf]

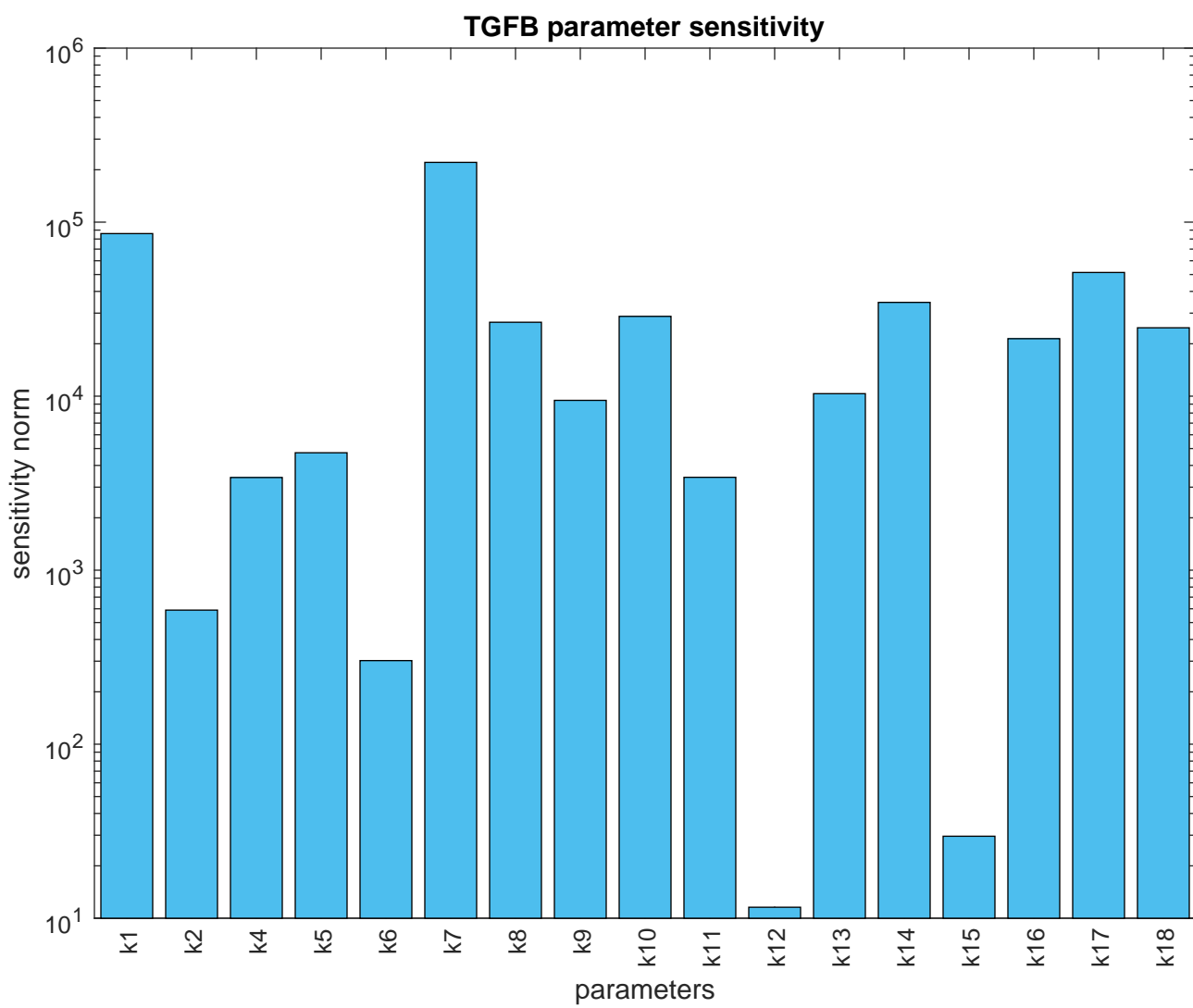

Supplement: Supplementary file 1 — VisId toolbox. This compressed folder contains the VisId MATLAB toolbox. (ZIP 1030 KB) [file 12918_2017_428_MOESM1_ESM.zip › visid-master/case_studies/tgfb/tgfb_sensitivity_bar.pdf]

**Circadian**  
**subset size dependence on collinearity threshold**

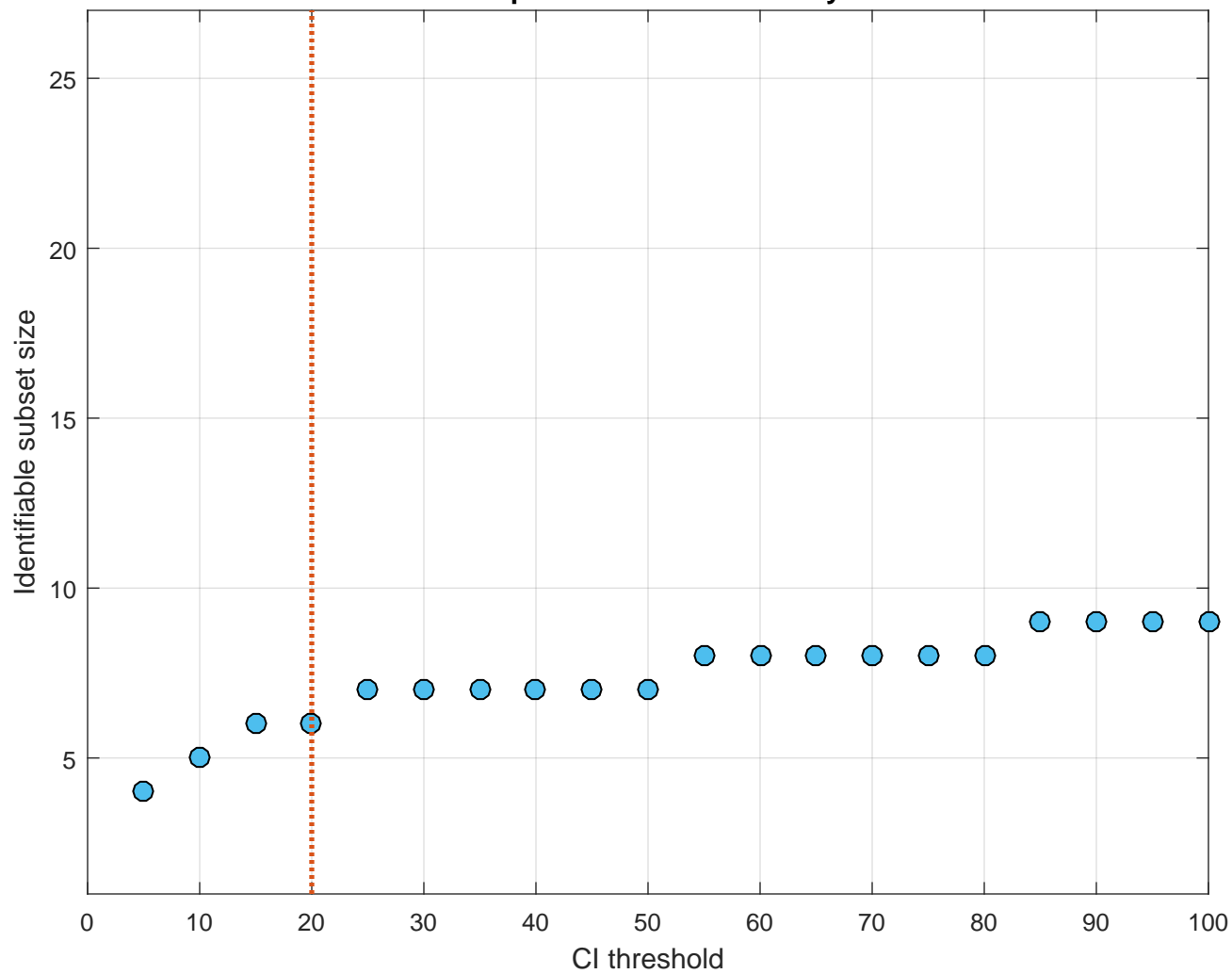

Supplement: Supplementary file 1 — VisId toolbox. This compressed folder contains the VisId MATLAB toolbox. (ZIP 1030 KB) [file 12918_2017_428_MOESM1_ESM.zip › visid-master/code/circadian/circadian_CI_idsubsetsize.pdf]

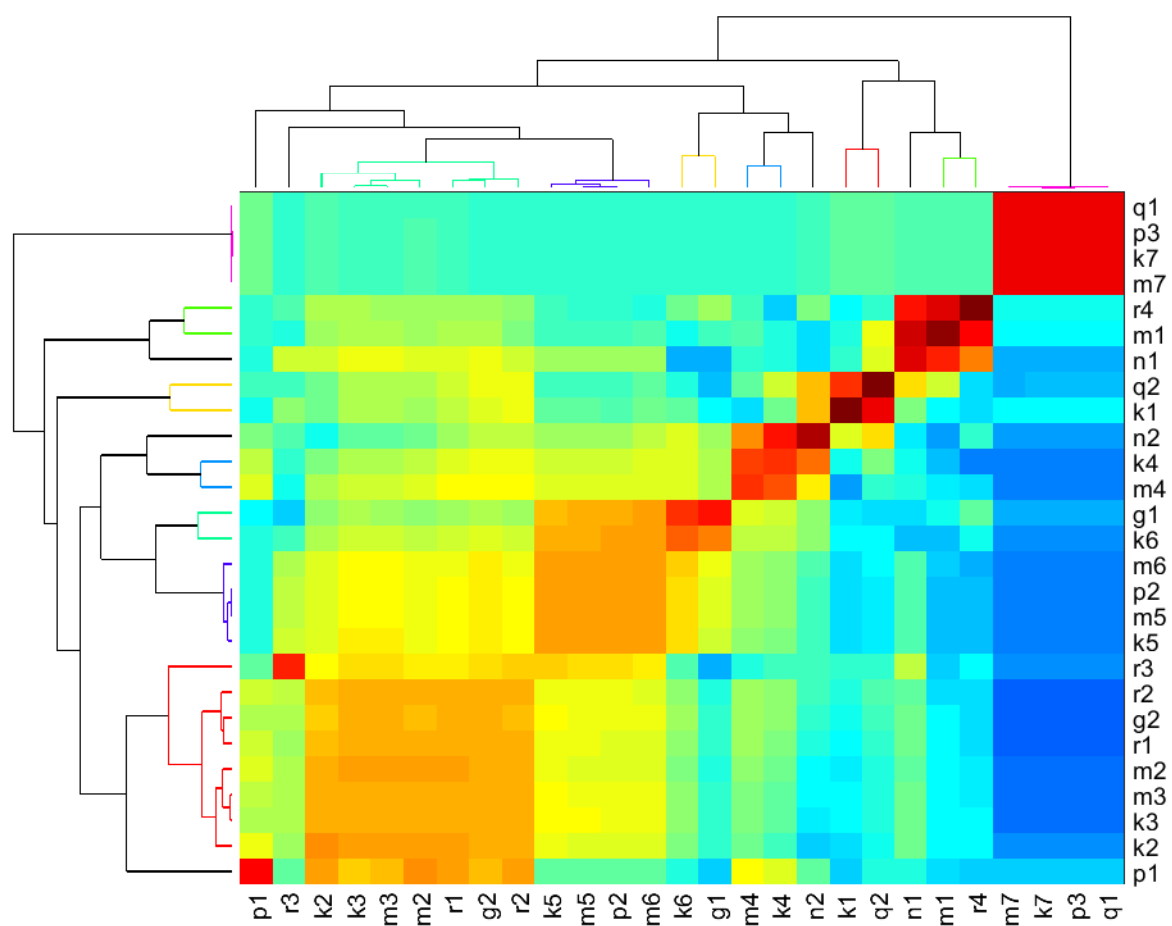

Supplement: Supplementary file 1 — VisId toolbox. This compressed folder contains the VisId MATLAB toolbox. (ZIP 1030 KB) [file 12918_2017_428_MOESM1_ESM.zip › visid-master/code/circadian/circadian_clustCorrmat.pdf]

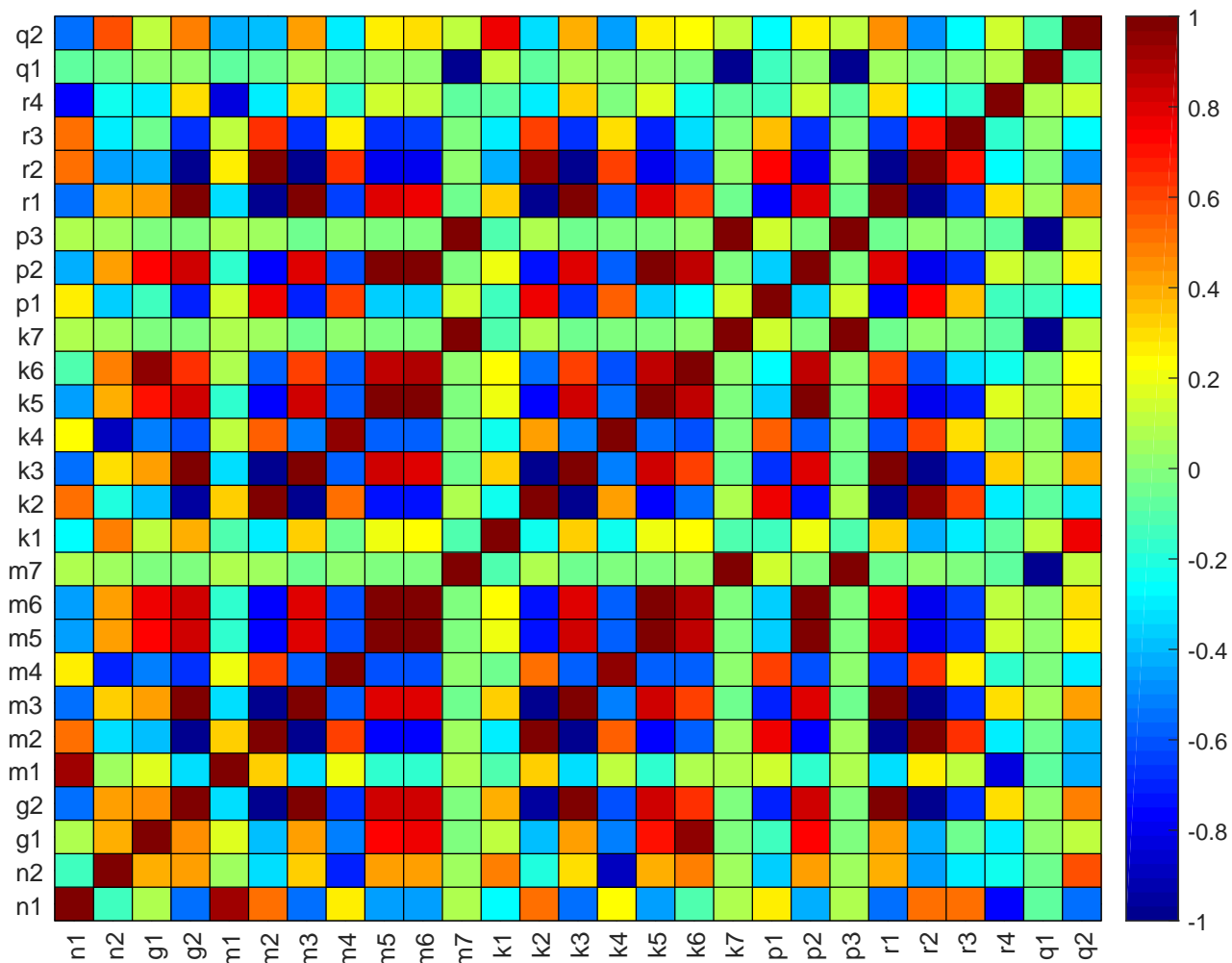

Supplement: Supplementary file 1 — VisId toolbox. This compressed folder contains the VisId MATLAB toolbox. (ZIP 1030 KB) [file 12918_2017_428_MOESM1_ESM.zip › visid-master/code/circadian/circadian_corrmat.pdf]

Circadian parameter sensitivity

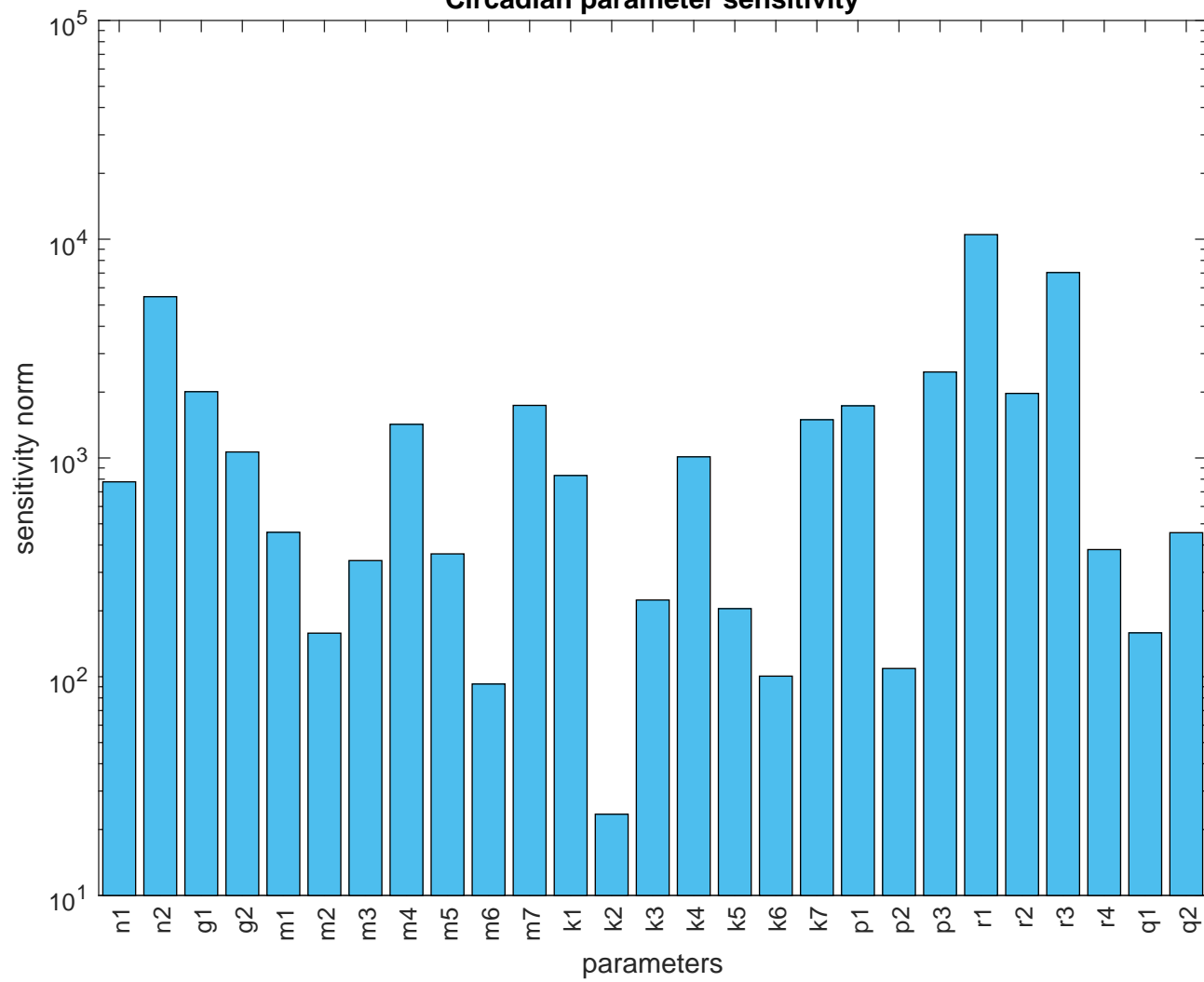

Supplement: Supplementary file 1 — VisId toolbox. This compressed folder contains the VisId MATLAB toolbox. (ZIP 1030 KB) [file 12918_2017_428_MOESM1_ESM.zip › visid-master/code/circadian/circadian_sensitivity_bar.pdf]
