# Supplementary material for: Parameter identifiability analysis and visualization in large-scale kinetic models of biosystems
Source: BMC Syst Biol. 2017 May 5;11:54. doi: 10.1186/s12918-017-0428-y (PMC5420165; doi:10.1186/s12918-017-0428-y)
Supplement: Supplementary file 2 — Supplementary material. This document contains detailed descriptions of the case studies and of the VisId toolbox, as well as additional details about the results. (PDF 306 KB) [file 12918_2017_428_MOESM2_ESM.pdf]

Supplementary material for “Parameter identifiability analysis and visualization in large-scale kinetic models of biosystems”

A. Gabor A.F. Villaverde J.R. Banga

March 13, 2017

Contents

1 Models 1
1.1 TGF-β pathway . . . 1
1.1.1 Equations . . . 1
1.1.2 Simulation details . . . 3
1.1.3 Parameters . . . 4
1.2 Circadian clock in *Arabidopsis Thaliana* . . . 6
1.2.1 Equations . . . 6
1.2.2 Parameters . . . 7

2 Identifiability results: computational cost 13

3 Optimization results: convergence curves 14

4 VisID Toolbox 16

1 Models

1.1 TGF-β pathway

1.1.1 Equations

This section shows the model equations of the TGF-β pathway, as presented in the work of Geier and coauthors [1]. The model includes 29 kinetic rates, which are as follows:

$$r_1 = k_1 C_{\text{TGFb\_TGFbR}}$$
$$r_2 = k_2 C_{\text{TGFbR}} C_{\text{TGFb}}$$
$$r_3 = k_3 C_{\text{TGFb\_TGFbR}} (1 - e^{-(\frac{t-k_{20}}{k_{21}})^{10}})$$
$$r_4 = k_4 C_{\text{TGFb\_TGFbR\_P}}$$
$$r_5 = k_5 C_{\text{TGFb\_TGFbR\_P}} C_{\text{I\_Smad}}$$
$$r_6 = k_6 C_{\text{I\_Smad\_TGFb\_TGFbR\_P}}$$
$$r_7 = k_7 C_{\text{Smad}} C_{\text{TGFb\_TGFbR\_P}}$$
$$r_8 = k_8 C_{\text{Smad}}$$
$$r_9 = k_9 C_{\text{Smad\_N}}$$
$$r_{10} = k_{10} 2 C_{\text{Smad\_P}} C_{\text{Smad\_P}}$$
$$r_{11} = k_{11} C_{\text{Smad\_P\_Smad\_P}}$$
$$r_{12} = k_{10} C_{\text{Smad\_P}} C_{\text{CoSmad}}$$
$$r_{13} = k_{11} C_{\text{Smad\_P\_CoSmad}}$$
$$r_{14} = k_8 C_{\text{CoSmad}}$$
$$r_{15} = k_9 C_{\text{CoSmad\_N}}$$
$$r_{16} = k_{12} k_8 C_{\text{Smad\_P\_Smad\_P}}$$
$$r_{17} = k_8 C_{\text{Smad\_P}}$$
$$r_{18} = k_9 C_{\text{Smad\_P\_N}}$$
$$r_{19} = k_{12} k_8 C_{\text{Smad\_P\_CoSmad}}$$
$$r_{20} = k_{13} C_{\text{Smad\_P\_N}}$$
$$r_{21} = k_{10} 2 C_{\text{Smad\_P\_N}} C_{\text{Smad\_P\_N}}$$
$$r_{22} = k_{11} C_{\text{Smad\_P\_Smad\_P\_N}}$$
$$r_{23} = k_{10} C_{\text{Smad\_P\_N}} C_{\text{CoSmad\_N}}$$
$$r_{24} = k_{11} C_{\text{Smad\_P\_CoSmad\_N}}$$
$$r_{25} = k_{14} \frac{C^2_{\text{Smad\_P\_CoSmad\_N}}}{C^2_{\text{Smad\_P\_CoSmad\_N}} + k^2_{15}}$$
$$r_{26} = k_{16} C_{\text{I\_Smad\_mRNA1}}$$
$$r_{27} = k_{17} C_{\text{I\_Smad\_mRNA2}}$$
$$r_{28} = k_{18} C_{\text{I\_Smad\_mRNA2}}$$
$$r_{29} = k_{19} C_{\text{I\_Smad}}$$

The kinetic rates appear in the differential equations of the 18 state variables, which represent the time-varying concentrations of the species involved in the pathway:

$$\begin{aligned}
\frac{dC_{\text{TGFb}}}{dt} &= r_1 - r_2\\
\frac{dC_{\text{dGFbR}}}{dt} &= r_1 - r_2\\
\frac{dC_{\text{TGFb_TGFbR}}}{dt} &= -r_1 + r_2 - r_3 + r_4 + r_6\\
\frac{dC_{\text{TGFb_TGFbR_P}}}{dt} &= r_3 - r_4 - r_5\\
\frac{dC_{\text{I_Smad_TGFb_TGFbR_P}}}{dt} &= r_5 - r_6\\
\frac{dC_{\text{Smad}}}{dt} &= -r_7 - r_8 + r_9\\
\frac{dC_{\text{Smad_P}}}{dt} &= r_7 - r_{10} + r_{11} - r_{12} + r_{13} - r_{17} + r_{18}\\
\frac{dC_{\text{CoSmad}}}{dt} &= -r_{12} + r_{13} - r_{14} + r_{15}\\
\frac{dC_{\text{Smad_P_Smad_P}}}{dt} &= r_{10} - r_{11} - r_{16}\\
\frac{dC_{\text{Smad_P_CoSmad}}}{dt} &= r_{12} - r_{13} - r_{19}\\
\frac{dC_{\text{Smad_N}}}{dt} &= r_8 - r_9 + r_{20}\\
\frac{dC_{\text{Smad_P_Smad_P_N}}}{dt} &= r_{16} + r_{21} - r_{22}\\
\frac{dC_{\text{Smad_P_N}}}{dt} &= r_{17} - r_{18} - r_{20} - r_{21} + r_{22} - r_{23} + r_{24}\\
\frac{dC_{\text{Smad_P_CoSmad_N}}}{dt} &= r_{19} + r_{23} - r_{24}\\
\frac{dC_{\text{CoSmad_N}}}{dt} &= r_{14} - r_{15} - r_{23} + r_{24}\\
\frac{dC_{\text{I_Smad_mRNA1}}}{dt} &= r_{25} - r_{26}\\
\frac{dC_{\text{I_Smad_mRNA2}}}{dt} &= r_{26} - r_{27}\\
\frac{dC_{\text{I_Smad}}}{dt} &= r_{28} - r_{29} - r_5 + r_6.
\end{aligned}$$

As in [1], we also assume that all the concentrations, except the Smad RNAs ($C_{\text{I\_Smad\_mRNA1}}$ and $C_{\text{I\_Smad\_mRNA2}}$), can be observed in the experiments.

1.1.2 Simulation details

Following the procedure described in [1], the initial conditions of the dynamic state variables were determined by finding their steady states. For this calculation, we took $C_{\mathrm{dGFbR}}(0) = 1$, $C_{\mathrm{Smad}}(0) = 60$ and $C_{\mathrm{CoSmad}}(0) = 60$, the initial concentrations of the other species as zero, and $k_3 = 0$ to temporarily remove the stimuli from the model. Then, simulations were performed for a suitable long time to obtain the steady state values of the variables. Finally, the value of C_{TGFb} was set to 1.0 and the nominal value (0.01) of k_3 was re-set. The numerical values of the steady state initial condition can be seen in Table 1.

Table 1: Nominal initial conditions for the TGF-β Pathway model

State name	Nominal initial condition
C_{TGFb}	1.0
C_{TGFbR}	1.0
$C_{\mathrm{TGFb\_TGFbR}}$	0.0
$C_{\mathrm{TGFb\_TGFbR_P}}$	0.0
$C_{\mathrm{I\_Smad\_TGFb\_TGFbR\_P}}$	0.0
C_{Smad}	40.98
$C_{\mathrm{Smad_P}}$	0.0
C_{CoSmad}	34.15
$C_{\mathrm{Smad\_P\_Smad\_P}}$	0.0
$C_{\mathrm{Smad\_P\_CoSmad}}$	0.0
$C_{\mathrm{Smad\_N}}$	19.02
$C_{\mathrm{Smad\_P\_Smad\_P\_N}}$	0.0
$C_{\mathrm{Smad\_P\_N}}$	0.0
$C_{\mathrm{Smad\_P\_CoSmad\_N}}$	0.0
$C_{\mathrm{CoSmad\_N}}$	15.85
$C_{\mathrm{I\_Smad\_mRNA1}}$	0.0
$C_{\mathrm{I\_Smad\_mRNA2}}$	0.0
$C_{\mathrm{I\_Smad}}$	0.0

The model equations were solved using the nominal parameters (see Table 2) and the nominal initial conditions (Table 1) for the time interval $t \in [0, 18000]$ seconds. The observation functions were evaluated at 15 time points equidistantly as $t = \mathrm{linspace}(0, 18000, 15)$ to obtain their nominal values. Pseudo-experimental data was generated using a standard deviation of 10% of the nominal signal level, while the detection thresholds for each observable was set to approximately 1% of their maximum level. This procedure generated 240 data points (16 observables, 15 time points per observable) for the model calibration.

1.1.3 Parameters

A list of the model parameters can be found in Table 2, which shows their name, nominal value, upper (UB) and lower (LB) bounds used for estimation purposes, and the estimated value obtained by optimization.

Table 2: TGF–β signalling pathway parameters

Par. id	Nominal	UB	LB	Estimated value
k_1	0.00015	0.1	10^{-6}	$1.6973 \cdot 10^{-4}$
k_2	0.023	1	0.0001	0.02524
k_3	0.01	not estimated		
k_4	0.01	1	10^{-6}	$9.089 \cdot 10^{-3}$
k_5	0.01	1	0.0001	$9.4683 \cdot 10^{-3}$
k_6	0.1	1	10^{-6}	0.10059
k_7	0.000404	1	10^{-6}	$3.9453 \cdot 10^{-4}$
k_8	0.0026	1	10^{-5}	$3.2970 \cdot 10^{-3}$
k_9	0.0056	1	10^{-5}	$7.0394 \cdot 10^{-3}$
k_{10}	0.002	1	10^{-6}	$2.14 \cdot 10^{-3}$
k_{11}	0.016	1	10^{-5}	0.016531
k_{12}	5.7	100	0.1	5.045
k_{13}	0.00657	1	10^{-5}	$7.06 \cdot 10^{-3}$
k_{14}	0.0017	1	10^{-5}	$1.4955 \cdot 10^{-3}$
k_{15}	1	100	0.001	1.3413
k_{16}	0.0008	0.1	10^{-5}	$8.8852 \cdot 10^{-4}$
k_{17}	0.001	0.1	10^{-5}	$8.9448 \cdot 10^{-4}$
k_{18}	0.0021	0.1	10^{-5}	2.0929
k_{19}	0.001	0.1	10^{-5}	8.1416
k_{20}	9000	not estimated		
k_{21}	1800			

All the identifiable subsets of model parameters can be found in table 3. We computed the subsets using collinearity threshold 20. The collinearity indices corresponding to each set (row) can be found in the second column.

Table 3: Identifiable subsets of TGF-β model parameters. Each row is a list of identifiable parameters. The collinearity indices corresponding to the sets are depicted in the second column (CI).

**Set**	**CI**	**Parameters**													
set 1	12.9	k1	k2	k4	k5	k6	k7	k8	k9	k10	k11	k13	k14	k15	k16
set 2	12.4	k1	k2	k4	k5	k6	k7	k8	k9	k10	k11	k13	k14	k15	k17
set 3	12.5	k1	k2	k4	k5	k6	k7	k8	k9	k10	k11	k13	k14	k15	k19
set 4	12.7	k1	k2	k4	k5	k6	k7	k8	k9	k10	k11	k13	k15	k16	k17
set 5	12.9	k1	k2	k4	k5	k6	k7	k8	k9	k10	k11	k13	k15	k16	k18
set 6	12.9	k1	k2	k4	k5	k6	k7	k8	k9	k10	k11	k13	k15	k16	k19
set 7	12.4	k1	k2	k4	k5	k6	k7	k8	k9	k10	k11	k13	k15	k17	k18
set 8	12.5	k1	k2	k4	k5	k6	k7	k8	k9	k10	k11	k13	k15	k18	k19
set 9	16	k1	k2	k4	k5	k6	k7	k8	k9	k10	k12	k13	k14	k15	k16
set 10	16	k1	k2	k4	k5	k6	k7	k8	k9	k10	k12	k13	k14	k15	k17
set 11	16	k1	k2	k4	k5	k6	k7	k8	k9	k10	k12	k13	k14	k15	k19
set 12	16	k1	k2	k4	k5	k6	k7	k8	k9	k10	k12	k13	k15	k16	k17
set 13	16	k1	k2	k4	k5	k6	k7	k8	k9	k10	k12	k13	k15	k16	k18
set 14	16	k1	k2	k4	k5	k6	k7	k8	k9	k10	k12	k13	k15	k16	k19
set 15	16	k1	k2	k4	k5	k6	k7	k8	k9	k10	k12	k13	k15	k17	k18
set 16	16	k1	k2	k4	k5	k6	k7	k8	k9	k10	k12	k13	k15	k18	k19
set 17	13.7	k1	k2	k4	k5	k6	k7	k8	k9	k11	k12	k13	k14	k15	k16
set 18	13.7	k1	k2	k4	k5	k6	k7	k8	k9	k11	k12	k13	k14	k15	k17
set 19	13.7	k1	k2	k4	k5	k6	k7	k8	k9	k11	k12	k13	k14	k15	k19
set 20	13.7	k1	k2	k4	k5	k6	k7	k8	k9	k11	k12	k13	k15	k16	k17
set 21	13.7	k1	k2	k4	k5	k6	k7	k8	k9	k11	k12	k13	k15	k16	k18
set 22	13.7	k1	k2	k4	k5	k6	k7	k8	k9	k11	k12	k13	k15	k16	k19
set 23	13.7	k1	k2	k4	k5	k6	k7	k8	k9	k11	k12	k13	k15	k17	k18
set 24	13.7	k1	k2	k4	k5	k6	k7	k8	k9	k11	k12	k13	k15	k18	k19
set 25	12.9	k1	k2	k4	k5	k6	k7	k8	k10	k11	k12	k13	k14	k15	k16
set 26	12.4	k1	k2	k4	k5	k6	k7	k8	k10	k11	k12	k13	k14	k15	k17
set 27	12.4	k1	k2	k4	k5	k6	k7	k8	k10	k11	k12	k13	k14	k15	k19
set 28	12.6	k1	k2	k4	k5	k6	k7	k8	k10	k11	k12	k13	k15	k16	k17
set 29	12.9	k1	k2	k4	k5	k6	k7	k8	k10	k11	k12	k13	k15	k16	k18
set 30	12.9	k1	k2	k4	k5	k6	k7	k8	k10	k11	k12	k13	k15	k16	k19
set 31	12.4	k1	k2	k4	k5	k6	k7	k8	k10	k11	k12	k13	k15	k17	k18
set 32	12.4	k1	k2	k4	k5	k6	k7	k8	k10	k11	k12	k13	k15	k18	k19
set 33	12.9	k1	k2	k4	k5	k6	k7	k9	k10	k11	k12	k13	k14	k15	k16
set 34	12.4	k1	k2	k4	k5	k6	k7	k9	k10	k11	k12	k13	k14	k15	k17
set 35	12.4	k1	k2	k4	k5	k6	k7	k9	k10	k11	k12	k13	k14	k15	k19
set 36	12.6	k1	k2	k4	k5	k6	k7	k9	k10	k11	k12	k13	k15	k16	k17
set 37	12.9	k1	k2	k4	k5	k6	k7	k9	k10	k11	k12	k13	k15	k16	k18
set 38	12.9	k1	k2	k4	k5	k6	k7	k9	k10	k11	k12	k13	k15	k16	k19
set 39	12.4	k1	k2	k4	k5	k6	k7	k9	k10	k11	k12	k13	k15	k17	k18
set 40	12.4	k1	k2	k4	k5	k6	k7	k9	k10	k11	k12	k13	k15	k18	k19

1.2 Circadian clock in *Arabidopsis Thaliana*

1.2.1 Equations

The model of the genetic network controlling the circadian clock in *Arabidopsis Thaliana* [2] is described by the following dynamic equations:

$$
\begin{aligned}
\frac{dCL_m}{dt} &= q1CP_n\theta_{light} + n1\frac{CT_n}{g1 + CT_n} - m1\frac{CL_m}{k1 + CL_m} \qquad (1)\\
\frac{dCL_c}{dt} &= p1CL_m - r1CL_c + r2CL_n - m2\frac{CL_c}{k2 + CL_c}\\
\frac{dCL_n}{dt} &= r1CL_c - r2CL_n - m3\frac{CL_n}{k3 + CL_n}\\
\frac{dCT_m}{dt} &= n2\frac{g2^2}{g2 + CL_n^2} - m4\frac{CT_m}{k4 + CT_m}\\
\frac{dCT_c}{dt} &= p2CT_m - r3CT_c + r4CT_n - m5\frac{CT_c}{k5 + CT_c}\\
\frac{dCT_n}{dt} &= r3CT_c - r4CT_n - m6\frac{CT_n}{k6 + CT_n}\\
\frac{dCP_n}{dt} &= (1 - \theta_{light})p3 - m7\frac{CP_n}{k7 + CP_n} - q2\theta_{light}CP_n
\end{aligned}
$$

1.2.2 Parameters

The nominal and the estimated model parameters can be found in Table 4.

Table 4: Arabidopsis Thaliana model parameters: nominal parameter values are used for generating the data, lower (LB) and upper (UB) bounds were used to estimate the parameter values.

Par.ID	Nominal Value	LB	UB	Estimated Value
n1	7.5038	0.0000	20.0000	7.3439
n2	0.6801	0.0000	20.0000	0.7944
g1	1.4992	0.0000	20.0000	1.4877
g2	3.0412	0.0000	20.0000	2.9788
m1	10.0982	0.0000	20.0000	10.4155
m2	1.9685	0.0000	20.0000	2.0399
m3	3.7511	0.0000	20.0000	2.7603
m4	2.3422	0.0000	20.0000	2.3275
m5	7.2482	0.0000	20.0000	7.3517
m6	1.8981	0.0000	20.0000	1.5123
m7	1.2000	0.0000	20.0000	1.1832
k1	3.8045	0.0000	20.0000	3.8762
k2	5.3087	0.0000	20.0000	9.8085
k3	4.1946	0.0000	20.0000	2.0002
k4	2.5356	0.0000	20.0000	2.0048
k5	1.4420	0.0000	20.0000	1.2678
k6	4.8600	0.0000	20.0000	0.9469
k7	1.2000	0.0000	20.0000	0.6841
p1	2.1994	0.0000	20.0000	2.1533
p2	9.4440	0.0000	20.0000	10.4056
p3	0.5000	0.0000	20.0000	0.6524
r1	0.2817	0.0000	20.0000	0.2965
r2	0.7676	0.0000	20.0000	0.9900
r3	0.4364	0.0000	20.0000	0.4424
r4	7.3021	0.0000	20.0000	7.4445
q1	4.5703	0.0000	20.0000	3.6431
q2	1.0000	0.0000	20.0000	0.2399

Small groups of colinear parameters are reported in table 5.

Table 5: *Arabidopsis Thaliana* model: highly colinear parameter sets. A set ID indicates the number of parameters involved in the colinearity group.

**Set ID.**	**CI**	**Parameters**		
G2(1)	34.1	n1	g1	
G2(2)	72.1	n1	m1	
G2(3)	43.7	n1	k1	
G2(4)	29	n1	r3	
G2(5)	40.4	n1	r4	
G2(6)	27	g1	m1	
G2(7)	77.4	g1	k1	
G2(8)	89.8	g1	r3	
G2(9)	98.5	g1	r4	
G2(10)	22	g2	p1	
G2(11)	23.9	g2	r1	
G2(12)	37.5	m1	k1	
G2(13)	22.9	m1	r3	
G2(14)	28.9	m1	r4	
G2(15)	554	m7	k7	
G2(16)	312	m7	p3	
G2(17)	172	m7	q1	
G2(18)	124	m7	q2	
G2(19)	46.3	k1	r3	
G2(20)	62.4	k1	r4	
G2(21)	200	k7	p3	
G2(22)	132	k7	q1	
G2(23)	158	k7	q2	
G2(24)	377	p3	q1	
G2(25)	89	p3	q2	
G2(26)	25	r1	r2	
G2(27)	99.5	r3	r4	
G2(28)	72.1	q1	q2	
G3(1)	35.6	n1	n2	m4
G3(2)	34.7	n1	n2	k4
G3(3)	21.5	n1	g2	m2
G3(4)	24.3	n1	g2	m3
G3(5)	21.9	n1	g2	k2
G3(6)	27.3	n1	g2	k3
G3(7)	21	n1	g2	r2
G3(8)	20.8	n1	m2	p1
G3(9)	21.4	n1	m2	r1
G3(10)	36.2	n1	m3	p1
G3(11)	24.5	n1	m3	r1

Continued on next page

Table 5 – continued from previous page

Set ID.	CI	Parameters		
G3(12)	21.5	n1	m3	r2
G3(13)	109	n1	m4	k4
G3(14)	39.4	n1	m5	p2
G3(15)	20.9	n1	m5	r2
G3(16)	23.1	n1	m6	r1
G3(17)	20.6	n1	m6	r2
G3(18)	20.6	n1	k2	r1
G3(19)	23.2	n1	k3	r1
G3(20)	31.4	n1	p1	r1
G3(21)	23.8	n1	p1	r2
G3(22)	20.4	n1	p2	r1
G3(23)	22.9	n1	p2	r2
G3(24)	32.5	n2	g1	m4
G3(25)	30.3	n2	g1	k4
G3(26)	55.3	n2	g2	m4
G3(27)	76.3	n2	g2	k4
G3(28)	103	n2	g2	r2
G3(29)	34.5	n2	m1	m4
G3(30)	32.5	n2	m1	k4
G3(31)	32	n2	m4	k1
G3(32)	20.8	n2	m4	k3
G3(33)	86.2	n2	m4	k4
G3(34)	33.9	n2	m4	p1
G3(35)	70.4	n2	m4	r1
G3(36)	65.8	n2	m4	r2
G3(37)	34.7	n2	m4	r3
G3(38)	34.3	n2	m4	r4
G3(39)	29.6	n2	k1	k4
G3(40)	20.4	n2	k2	k3
G3(41)	21.5	n2	k3	p1
G3(42)	32.1	n2	k4	p1
G3(43)	126	n2	k4	r1
G3(44)	96.1	n2	k4	r2
G3(45)	33	n2	k4	r3
G3(46)	32.7	n2	k4	r4
G3(47)	23.8	n2	p1	r2
G3(48)	20.3	g1	g2	m2
G3(49)	25.7	g1	g2	m3
G3(50)	20.8	g1	g2	k3
G3(51)	25.2	g1	m3	p1
G3(52)	21	g1	m3	r1
G3(53)	99.2	g1	m4	k4
G3(54)	20.2	g1	m5	r1

Continued on next page

Table 5 – continued from previous page

Set ID.	CI	Parameters		
G3(55)	21.4	g1	m6	r1
G3(56)	28.2	g1	p1	r1
G3(57)	23.5	g1	p2	r1
G3(58)	21.8	g2	m1	m3
G3(59)	20.1	g2	m1	k2
G3(60)	24.4	g2	m1	k3
G3(61)	21	g2	m1	r2
G3(62)	25.6	g2	m2	r2
G3(63)	23.6	g2	m2	r3
G3(64)	23.3	g2	m2	r4
G3(65)	23.9	g2	m3	k1
G3(66)	22.2	g2	m3	r2
G3(67)	31.2	g2	m3	r3
G3(68)	29.8	g2	m3	r4
G3(69)	131	g2	m4	k4
G3(70)	86.2	g2	m4	r2
G3(71)	32.1	g2	m5	k6
G3(72)	20.5	g2	m7	r2
G3(73)	20.5	g2	k1	k3
G3(74)	21.2	g2	k2	k3
G3(75)	53.1	g2	k2	r2
G3(76)	20.2	g2	k2	r4
G3(77)	46.3	g2	k3	r2
G3(78)	23	g2	k3	r3
G3(79)	24.1	g2	k3	r4
G3(80)	93	g2	k4	r2
G3(81)	28.6	g2	k5	r2
G3(82)	20.4	g2	k7	r2
G3(83)	20.6	g2	p3	r2
G3(84)	20.8	g2	r2	q1
G3(85)	20.1	g2	r2	q2
G3(86)	32.6	m1	m3	p1
G3(87)	22.1	m1	m3	r1
G3(88)	20.5	m1	m3	r2
G3(89)	98.8	m1	m4	k4
G3(90)	39.2	m1	m5	p2
G3(91)	20.5	m1	m5	r2
G3(92)	22.3	m1	m6	r1
G3(93)	20.3	m1	m6	r2
G3(94)	21.2	m1	k3	r1
G3(95)	29.6	m1	p1	r1
G3(96)	23.7	m1	p1	r2
G3(97)	22.9	m1	p2	r2

Continued on next page

Table 5 – continued from previous page

Set ID.	CI	Parameters		
G3(98)	33.9	m2	m3	p1
G3(99)	23.8	m2	m3	r1
G3(100)	34.8	m2	m3	r2
G3(101)	27	m2	m4	k4
G3(102)	47.2	m2	p1	r1
G3(103)	157	m2	p1	r2
G3(104)	20.5	m2	r1	r3
G3(105)	20.9	m2	r1	r4
G3(106)	21.2	m3	m4	k3
G3(107)	48.2	m3	m4	k4
G3(108)	25.9	m3	k1	p1
G3(109)	20.7	m3	k1	r1
G3(110)	23.4	m3	k2	k3
G3(111)	23.5	m3	k3	k4
G3(112)	68.7	m3	p1	r1
G3(113)	81.5	m3	p1	r2
G3(114)	26.8	m3	p1	r3
G3(115)	29.5	m3	p1	r4
G3(116)	23.6	m3	r1	r3
G3(117)	24.4	m3	r1	r4
G3(118)	35.5	m4	m5	k4
G3(119)	20.6	m4	m6	k4
G3(120)	68.3	m4	m7	k4
G3(121)	95.9	m4	k1	k4
G3(122)	59.5	m4	k2	k4
G3(123)	60.4	m4	k3	k4
G3(124)	21.7	m4	k4	k6
G3(125)	68.4	m4	k4	k7
G3(126)	106	m4	k4	p1
G3(127)	68.2	m4	k4	p3
G3(128)	114	m4	k4	r1
G3(129)	113	m4	k4	r2
G3(130)	104	m4	k4	r3
G3(131)	105	m4	k4	r4
G3(132)	68.2	m4	k4	q1
G3(133)	68	m4	k4	q2
G3(134)	21.6	m4	p1	r2
G3(135)	20.7	m5	k1	p2
G3(136)	22.7	m5	k6	p1
G3(137)	21.5	m5	k6	r1
G3(138)	21.1	m5	p2	r4
G3(139)	20.4	m5	r1	r3
G3(140)	21.8	m6	k1	r1

Continued on next page

Table 5 – continued from previous page

Set ID.	CI	Parameters		
G3(141)	21.2	m6	r1	r3
G3(142)	21.5	m6	r1	r4
G3(143)	28.2	k1	p1	r1
G3(144)	23.2	k1	p2	r1
G3(145)	20.1	k2	k3	k5
G3(146)	22.5	k2	k3	p1
G3(147)	21.5	k2	k3	r1
G3(148)	21.8	k2	k3	r2
G3(149)	24.3	k2	p1	r1
G3(150)	41.2	k2	p1	r2
G3(151)	25.3	k3	p1	r1
G3(152)	45	k3	p1	r2
G3(153)	20.6	k3	r1	r4
G3(154)	21.5	k4	p1	r2
G3(155)	31.1	p1	r1	r3
G3(156)	32.1	p1	r1	r4
G3(157)	22.7	p2	r1	r3
G3(158)	22.1	p2	r1	r4

2 Identifiability results: computational cost

The table below contains the average CPU time (in seconds) required by the key computational procedures of the identifiability methodology for each of the case studies presented in the main paper. The calculations were done on a MacBook Pro 2015.

Table 6: Computational performance: CPU time (in seconds). The meaning of each column is explained below the table.

**Case**	**SM**	**pairwise CI**	**triplet CI**	**Largest set**	**All largest sets**
TGF-β	0.282	0.012 ± 0.007	0.035 ± 0.0077	2.038 ± 0.393	7.589 ± 0.274
Circadian	0.511	0.012 ± 0.0018	1.260 ± 0.084	1.162 ± 0.092	1.709 ± 0.057
B2	0.780	0.083 ± 0.0046	8.030 ± 0.371	22.682 ± 1.175	-
B4	5.124	0.105 ± 0.026	302.268 ± 46.131	2.909 ± 0.793	-

Column 1: case study.
Column 2: calculating the sensitivity matrix (SM).
Column 3: calculating the collinearity index of pairs of parameters.
Column 4: calculating the collinearity index of triplets of parameters.
Column 5: finding the largest identifiable subset.
Column 6: finding all the possible largest identifiable subsets.

3 Optimization results: convergence curves

This section presents the convergence curves of the optimization algorithms used for the parameter estimation of models B2 and B4. Figures 1 and 2 show the convergence curves, i.e. the current best objective function value against the CPU time during the optimization of the models, of the calibration of the models B2 and B4, respectively. Here, three methods were compared: the global optimization method enhanced scatter search (eSS) is combined with the adaptive least squares solver (NL2Sol) or with fmincon (from MATLAB). In case of 'eSS-NL2Sol reg', we solved the regularized parameter estimation problem, which prevents over-fitting. We ran each method 5 times, independently, using different initial guesses of parameters and different random seeds for the random number generator. In the figures we depicted the best performing cases for each method.

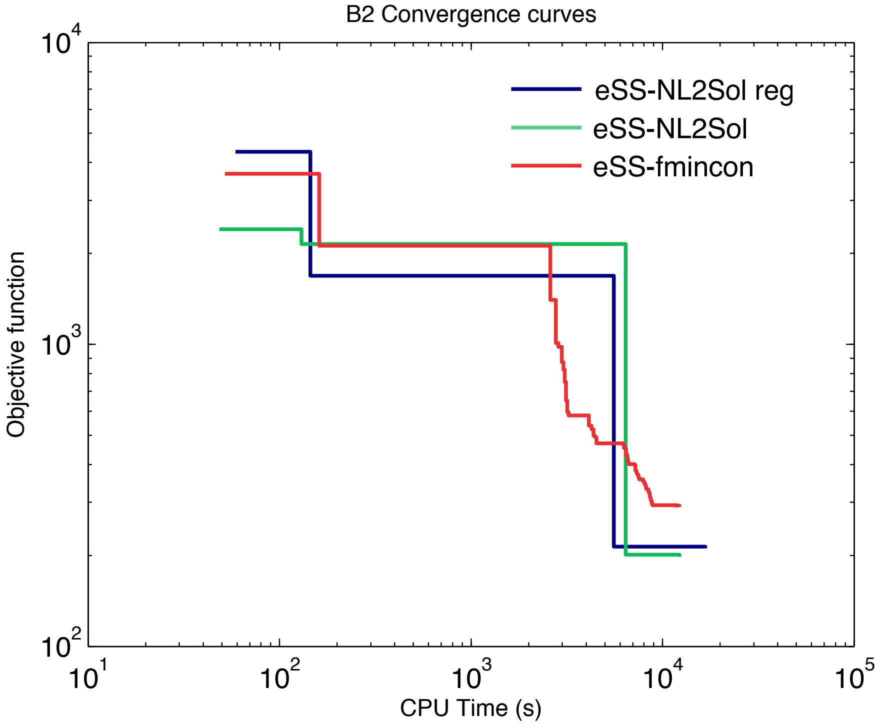

Figure 1: Convergence curves of the calibration of model B2. The best convergence curves out of 5 runs are depicted for the 3 compared methods.

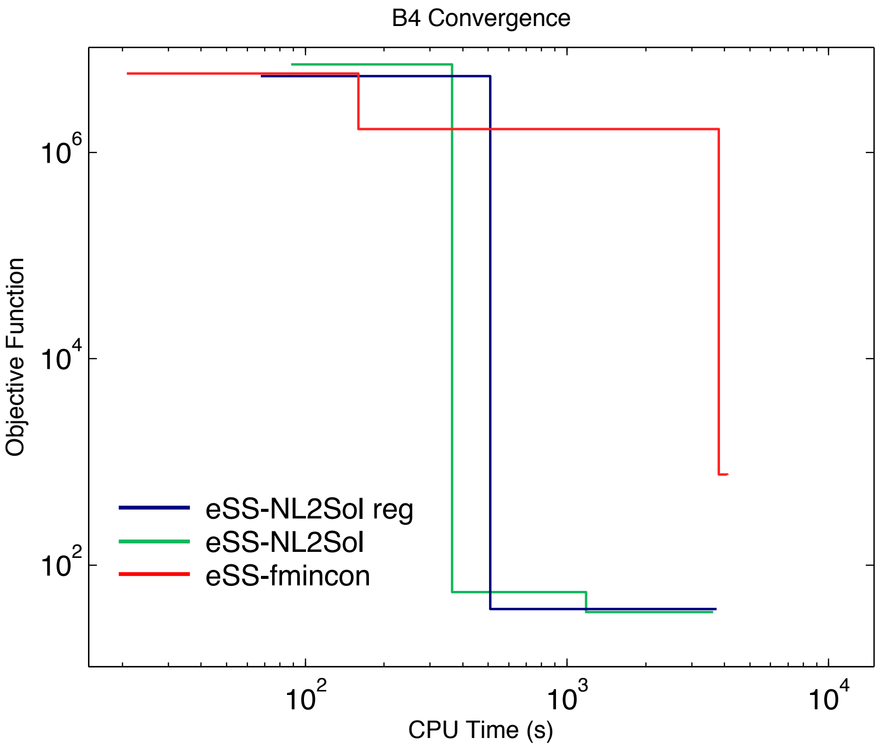

Figure 2: Convergence curves of the calibration of model B4. The best convergence curves out of 5 runs are depicted for the 3 compared methods.

4 VisID Toolbox

The VisID MATLAB Toolbox can be downloaded from the following GitHub repository: `https://github.com/gabora/visid`.

The Toolbox provides the practical identifiability analysis of model parameters after a parameter estimation was performed.

Software Requirements.

- MATHWORKS MATLAB (`https://www.mathworks.com/products/matlab.html`)
- AMIGO2 (`https://sites.google.com/site/amigo2toolbox/download`) is used to build, simulate and calibrate the models using experimental data.
- A distribution of MEIGO Toolbox (`http://www.iim.csic.es/~gingproc/meigo.html`), which contains an implementation of Variable Neighboring Search (VNS) integer optimisation algorithm, is included in the package, but the latest version can be downloaded from the above link.
- Rank revealing QR algorithm can be downloaded from `https://www.mpi-magdeburg.mpg.de/1094756/rrqr`. In case the algorithm is not available for certain operation system, then the default QR decomposition of MATLAB is used. We found that the built-in QR algorithm works less efficiently for the initialization of finding the largest identifiable parameter set.
- Cytoscape (`http://www.cytoscape.org/`) for visualisation of the networks.

Inputs for parameter estimation. The parameter estimation was performed in AMIGO2 Toolbox (`https://docs.google.com/viewer?a=v&pid=sites&srcid=ZGVmYXVsdGRvbWFpbnxhbWlnbzJ0b29sYm94fGd4OmRmZTg3NDg1OTIyNDc1OA`). The basic inputs for parameter estimation are

- model equations
- observation equations
- known model parameters
- initial conditions for the state variables
- stimuli profile (if any stimulus applied)
- experimental dataset (time-series)
- set of estimated parameters

- initial guess of model parameters (optional)
- upper and lower bounds for estimated parameters
- settings for the integrator (CVODES) and optimsation algorithm (eSS and NL2SOL)

Examples for defining the parameter estimation can be found in the case_studies folder of the VisID toolbox, and in the examples provided with the AMIGO2 toolbox `https://sites.google.com/site/amigo2toolbox/examples`.

Inputs for VisID Toolbox. VisID toolbox performs the following tasks

Task1 determines the largest identifiable subset (non-unique)

Task2 determines all the largest subsets

Task3 determines small collinear groups of parameters up to a set of K parameter

Task4 visualizes collinear groups of parameters to Cytoscape

Task5 visualizes the network with identifiable/non-identifiable parameters in Cytoscape.

Task1-Task4 are sensitivity based calculations and only the estimated parameters and the Jacobian matrix of the residual vector ($\frac{\partial R}{\partial \theta}(\hat{\theta})$) is required. This is part of the output structure of the parameter estimation task of AMIGO2. *Task5* further requires that the model equations are given in the AMIGO2 input format (as character arrays).

Outputs of VisID Toolbox. Depending on which of the above task is evaluated, VisID generates LaTeXtables for collinear groups of parameters and for all the largest identifiable sets. Further it generates network and edge files, which can be imported in Cytoscape for visualization.

Known limitations. We found that Task 1 works very well even on large scale models. It was performed for the model B1 in [3] (not reported in this manuscript), which contains 1759 parameters. The QR decomposition took approximately 20 minutes on a DELL Workstation Precision. However, in this case memory can be a limiting factor for computations performed on regular PCs.

Task 2 scales exponentially with the number of parameters, since it requires the computation of collinearity indexes for all groups of parameters, thus the model size can be a limiting factor for these calculations.

Task 3 depends on the parameter K and number of parameters. In case there are N parameters, the brute force approach performs $\sum_{i=2}^{K} \binom{N}{i}$ singular value decompositions to estimate the collinearity indexes. Therefore collinear pairs can be always evaluated, triplets are also possible for models with approximately

less than 100 parameters. For medium size models, for example for the TGF-β case study we were able to compute collinearity groups of up to 6 parameters in a few minutes.

References

[1] Geier, F., Fengos, G., Felizzi, F., Iber, D.: Analyzing and Constraining Signaling Networks: Parameter Estimation for the User. In: Liu, X., Betterton, M.D. (eds.) Computational Modeling of Signaling Networks. Methods in Molecular Biology, vol. 880, pp. 23–40. Humana Press, Totowa, NJ (2012). doi:10.1007/978-1-61779-833-7. http://www.springerlink.com/index/10.1007/978-1-61779-833-7

[2] Locke, J.C.W., Millar, a.J., Turner, M.S.: Modelling genetic networks with noisy and varied experimental data: the circadian clock in Arabidopsis thaliana. Journal of Theoretical Biology **234**(3), 383–393 (2005). doi:10.1016/j.jtbi.2004.11.038

[3] Villaverde, A.F., Henriques, D., Smallbone, K., Bongard, S., Schmid, J., Cicin-Sain, D., Crombach, A., Saez-Rodriguez, J., Mauch, K., Balsa-Canto, E., Mendes, P., Jaeger, J., Banga, J.R.: BioPreDyn-bench: a suite of benchmark problems for dynamic modelling in systems biology. BMC Systems Biology **9**(1), 8 (2015). doi:10.1186/s12918-015-0144-4
